# The Conservation Genetics of *Iris lacustris* (Dwarf Lake Iris), a Great Lakes Endemic

**DOI:** 10.3390/plants12132557

**Published:** 2023-07-05

**Authors:** James Isaac Cohen, Salomon Turgman-Cohen

**Affiliations:** 1 Department of Botany and Plant Ecology, Weber State University, 1415 Edvalson St., Dept. 2504, Ogden, UT 84408-2504, USA; 2 E.S. Witchger School of Engineering, Marian University, 3200 Cold Spring Road, Indianapolis, IN 46222-1997, USA; sturgmancohen@marian.edu

**Keywords:** genotyping-by-sequencing, *Iris*, Lake Huron, Lake Michigan, polyploidy, polyRAD, rare plants, tGBS

## Abstract

*Iris lacustris*, a northern Great Lakes endemic, is a rare species known from 165 occurrences across Lakes Michigan and Huron in the United States and Canada. Due to multiple factors, including habitat loss, lack of seed dispersal, patterns of reproduction, and forest succession, the species is threatened. Early population genetic studies using isozymes and allozymes recovered no to limited genetic variation within the species. To better explore genetic variation across the geographic range of *I. lacustris* and to identify units for conservation, we used tunable Genotyping-by-Sequencing (tGBS) with 171 individuals across 24 populations from Michigan and Wisconsin, and because the species is polyploid, we filtered the single nucleotide polymorphism (SNP) matrices using polyRAD to recognize diploid and tetraploid loci. Based on multiple population genetic approaches, we resolved three to four population clusters that are geographically structured across the range of the species. The species migrated from west to east across its geographic range, and minimal genetic exchange has occurred among populations. Four units for conservation are recognized, but nine adaptive units were identified, providing evidence for local adaptation across the geographic range of the species. Population genetic analyses with all, diploid, and tetraploid loci recovered similar results, which suggests that methods may be robust to variation in ploidy level.

## 1. Introduction

In 1818, Thomas Nuttall described a new species of crested *Iris* L., *Iris lacustris* Nutt., “on the gravelly shores of calcareous islands of Lake Huron” [1]. Since then, the recognized geographic range of the species has expanded to include the northern regions of Lakes Huron and Michigan in the United States and Canada. Presently, the species is known from 165 occurrences, with more than half in Michigan (89) and the others split between Wisconsin (36) and Ontario (40) [2].

**Figure 1:**
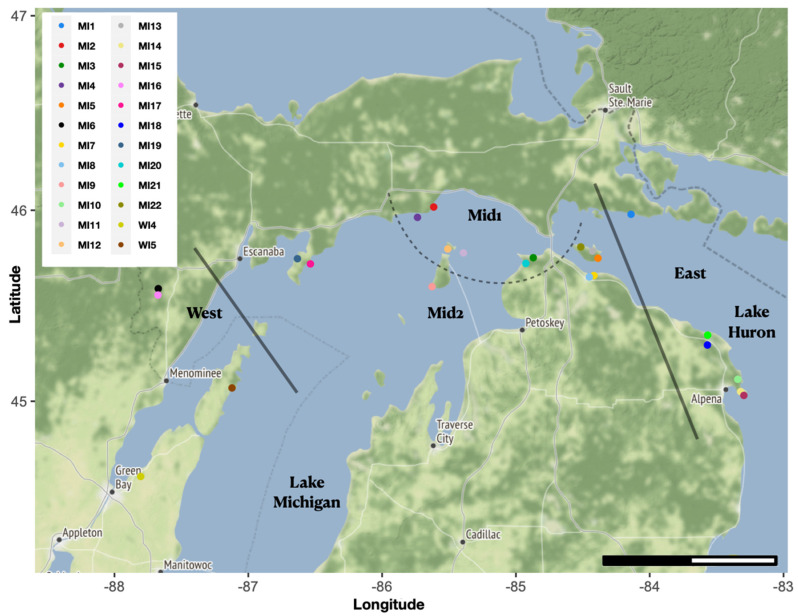
Map of locations sampled in present study. Dark gray entire lines denote division between East, Mid1, Mid2, and West clusters (also recognized as management units). The dashed gray line separates Mid1 and Mid2 populations, and Mid includes both groups of populations together. Light gray lines separate Wisconsin (USA), Michigan (USA), and Ontario (Canada). Scale bar is 100 km, with each section representing 50 km.

Plants of *I. lacustris* grow less than 15 cm in height [3], and this feature provides the species with its common name, Dwarf Lake Iris. The species bears self-compatible flowers, with purple sepals and purple petals with yellow and white markings, that are visited by various species of bees [4]. Across its geographic range, *I. lacustris* frequently inhabits the understory of coniferous forests along the shore, although a small number of inland populations are known (Figure 1) [2,5,6]. These habitats have thin entisols, and the dominant tree species primarily include *Thuja occidentalis* L., *Abies balsamea* (L.) Miller, and *Picea glauca* (Moench) Voss. The species has become a well-known endemic plant of the Great Lakes and is so characteristic of the region that it was recognized as the state wildflower of Michigan [7].

In 1988, 170 years after *I. lacustris* was initially described, the species was listed as federally threatened [5]. The small number of populations and individuals is due to multiple factors, including the loss of shoreline habitat, fungal infection of fruits, lack of seed dispersal, and overgrowth of the forest canopy that restricted plant growth, flower production, and sexual reproduction. Plants of the species currently reproduce more by vegetative growth than germination from the myrmecochorous seeds [5]. Despite this low germination rate, seeds can remain viable in the seedbank for at least 15 years [5], a factor that could influence long-term population growth and genetic diversity, although mass germination and recruitment are rare [4].

The ecology of *I. lacustris* has been examined to a greater extent than the genetic diversity of the species. To date, only three studies have explored this topic: Simonich and Morgan [8] examined nine populations in Wisconsin, using 22 allozyme markers, Orick [9] investigated nine populations in Michigan, using 24 isozymes, and Hannan and Orick [10] examined nine populations in Michigan, using 18 isozymes. In two studies, researchers identified genetic homogeneity across the populations; however, Orick [9] found overall heterozygosity to be 3.7%. Hannan and Orick [10] also note gene silencing may have been possible in four loci. In contrast to the genetic diversity recognized in *I. lacustris*, Hannan and Orick [10] found that the sister species, *I. cristata* Aiton [11], which has a wider geographic range across eastern North America, was variable at 11 of 15 loci. These studies suggest that the genetic diversity of this rare species of *Iris* is quite limited. This genetic paucity is intriguing because *I. lacustris* and its sister are both putative tetraploids [10], and polyploid plant species tend to have greater genetic variation than diploid relatives, although selfing tends to be higher in polyploids [12,13,14]. Importantly, the genetic diversity of the *I. lacustris* may have implications for the ability of the species to respond to the changing environment across its geographic range and for various conservation efforts.

In order to investigate the population and conservation genetics of the species in a comprehensive manner, we examined multiple populations from across Michigan and Wisconsin, and we used tunable Genotyping-by-Sequencing (tGBS [15]), a method of reduced representation sequencing, to identify single nucleotide polymorphisms (SNPs) among the populations. The objectives of the present study are threefold: (1) identify genetic diversity and population structure and substructure across the range of *I. lacustris*, (2) explore patterns of migration, and (3) recognize population clusters for management of this rare species. Given the paucity of genetic diversity identified in previous studies, we hypothesized that there would be limited genetic variation across the species.

## 2. Results

### 2.1. DNA Sequencing and Polyploid Filtering

Among the 171 individuals of 24 populations across the geographic range of *I. lacustris* in Michigan and Wisconsin (Figure 1, Table 1), 726,786,603 paired-end reads were sequenced, with a mean of 4,225,503 reads per sample. The consensus sequence included 1,335,996 scaffolds with 196,139,854 bp (N50 = 644,994, L50 = 145). The mean per sample alignment and unique alignment to the consensus sequences are 93.9% and 74.4%, respectively. For the MCR90 dataset, 125 reads were interrogated per SNP across 2,341,730 bases, with 4.8% missing data for the final dataset. For the MCR50 dataset, 31 reads were interrogated per SNP across 23,904,409 bases, with 31.4% missing data for the final dataset. The numbers of SNPs in the diploid and tetraploid datasets identified through analysis in polyRAD are in Table 2.

### 2.2. Population Genomics

Across all datasets, observed heterozygosity slightly exceeds expected heterozygosity, and F_IS_ values are, in general, negative (Table 3). Pairwise F_ST_ values vary from 0.1–0.45, and results are similar among datasets (Table 4). Based on various AMOVA results, most of the variation is within samples, followed by between the populations, regardless of the datasets and partitioning of the populations (Supplemental Table S1). Mantel tests for isolation-by-distance analyses identify all datasets as having spatial structure (Supplemental Figure S1) with *p* < 0.001 for analyses of individuals, but only MCR90 datasets had spatial structure for populations (*p* < 0.05).

**Figure 2:**
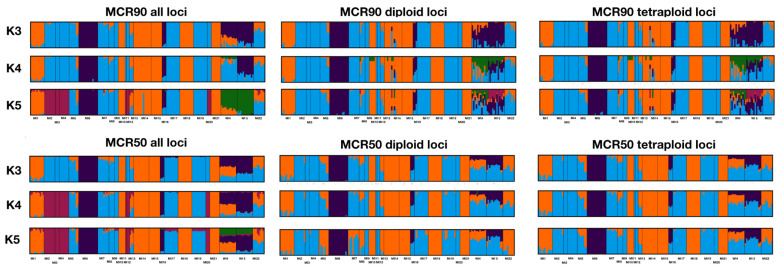
Structure bar graphs from fastSTRUCTURE for the six datasets analyzed in the present study for K = 3–5. Individual ancestry denoted by color.

Results from analyses in fastSTRUCTURE, STRUCTURE, MavericK, and tess3r are similar. Based on the results from StructureSelector, the optimal K values were greater for all loci analyzed together than for either the diploid or tetraploid loci analyzed independently (Table 2, Supplemental Table S2). Similar clusters were recovered with the different datasets (Figure 2, Table 1), with a clear division between three groups—eastern, western, and central populations—and multiple analyses resulted in the central population being divided into two distinct groups at K = 4 and/or 5 (Figure 1, Supplemental Figures S2–S4), especially for all loci in fastSTRUCTURE and multiple datasets with STRUCTURE, MavericK, and tess3r. At K = 4–5, the two Wisconsin populations were often recovered with unique genomic signatures suggestive of admixture, and this is particularly the case with the MCR90 datasets. While the results of conStruct are similar to others, the three distinct groups identified are more opaque, with boundaries between the eastern and western populations overlapping to a larger extent than with the other analyses (Supplemental Figure S5); although, similar patterns can be recognized at K = 4 and 5 for the MCR90 all and diploid loci datasets. Among all methods, the three populations on Bois Blanc Island in Michigan (MI5, MI13, and MI22), in the northwestern geographic range of the species, also include some individuals that show signals of admixture between the eastern and central populations (Figure 2). Graphs of K values for all analyses are included in Supplementary Materials (Figures S6–S16).

The results of principal components analysis (PCA) and discriminant analyses of principal components (DAPC) are similar to those that explicitly consider a priori population structure. With PCA, three to four clusters were recovered corresponding to the same ones from the population assignation analyses, and this was more evident with the MCR90 datasets compared to the MCR50 ones. In all analyses, three populations—MI6, MI16, and WI5—were recognized as most distinct from the other populations. Across DAPC analyses, individuals from populations tended to cluster together, and this is similar to results from other methods. In general, DAPC analyses recover MI6, MI16, and WI5 as distinct units or as a cluster together, with the results for MCR50 all loci being the only exception. In analyses with this dataset, WI5 was included in a cluster distinct from the other two populations, but with WI4 and populations from Michigan. In some analyses, such as MCR50 and MCR90 diploid loci, the divided cluster of central populations was identified. The number of loci under selection in each dataset is in Table 2.

**Figure 3:**
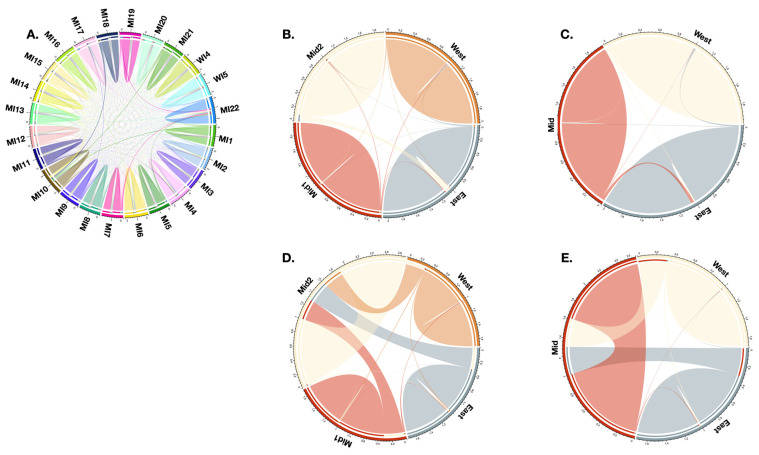
Patterns of migration based on MCR90 all loci (**A**–**C**) and MCR90 diploid loci (**D**,**E**) as resolved using BA3-SNPs. (**A**) All populations, (**B**,**D**) 4 populations, (**C**,**E**) 3 populations. Outermost circle denotes each population, and inner circle shows origin of migrants from each population. Lines connecting populations demonstrate patterns of migration.

Patterns of migration inferred from BA3-SNPs suggest that migration is minimal, regardless of the dataset analyzed, and that most individuals are from their original population (Figure 3). While this was certainly the case for all loci for MCR90, analyses with only the diploid loci for three or four population clusters (Table 1) provide evidence of greater rates of migration between adjacent populations (Figure 3). Migration directly between the eastern and western populations was negligible. The relationship among the four population clusters that was most supported by the results of DIYABC-RF and abcranger varies depending on the dataset analyzed. For all, diploid, and tetraploid loci, (West (Mid1 (Mid2, East))), (West (East (Mid1, Mid2))), and (West (Mid1 (Mid2, East))) are recovered as optimal, respectively, and (Mid2 (West (Mid2, East))) and (West (East (Mid1, Mid2))) are identified as close second choices for all and tetraploid datasets, respectively. The one constant among the three optimal trees is that the western population is recognized to have diverged prior to the mid and eastern populations, and this also is the case for one of the near-optimal trees (Supplemental Figure S17).

### 2.3. Conservation Units

**Figure 4:**
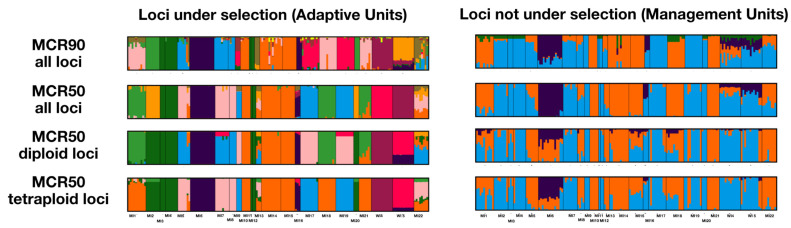
Structure bar graphs for MCR90 all loci and three MCR50 datasets for loci under and not under selection (adaptive units and management units, respectively). Individual ancestry denoted by color. Groups for each listed in Table 1, and best K values noted in Table 2.

Based on the method of Funk et al. [16], evolutionarily significant units (ESUs) were identified using all loci, as described below, and the management units (MUs), which are based on fastSTRUCTURE, PCA, and DAPC analyses with loci not under selection, are quite similar. The largest difference between ESUs and MUs is that the two populations in Wisconsin may or may not be included with the other two western populations, MI6 and MI16, depending on the use of all loci or only diploid or tetraploid loci (Figure 4). The populations on Bois Blanc Island also have mixed ancestry based on these loci. The adaptive units, which are based on fastSTRUCTURE, PCA, and DAPC analyses with loci under selection, provide quite different results. Generally, among analyses, nine adaptive units are recognized, and these are structured based on geography (Figure 4, Supplemental Figure S18, Table 1).

The results of the fastSTRUCTURE, PCA, and DAPC are similar, with one exception. Unlike analyses with fastSTRUCTURE and PCA, where individuals of the same population cluster together, with DAPC, some individuals of the same population are members of different clusters. This is likely due to the large number of clusters identified as optimal, which is particularly the case for MCR90 and MCR50 datasets with all loci.

## 3. Discussion

### 3.1. Population Structure and Genetic Diversity

Based on the multiple datasets explored using various methodological approaches, three or four different population clusters were frequently recognized for *I. lacustris* across Michigan and Wisconsin. These clusters are structured geographically, with eastern, central, and western groups, and at higher K values, the central group is subdivided into two groups that are also geographically oriented (Figure 1 and Figure 2). In the three prior studies that employed isozymes and allozymes to examine the population genetics of *I. lacustris* [8,9,10], no to limited genetic diversity was identified in the populations. Each study only investigated the genetic diversity of populations within one state, using markers available at the time, which likely led to the paucity of genetic diversity. In the present study, many more loci were examined, and individuals from across most of the geographic range of the species were analyzed together, which provides a more holistic approach to elucidating the genetic diversity of the species. These results demonstrate that our hypothesis—a lack of genetic diversity among the species—was incorrect.

Across all studied populations, statistically significant isolation-by-distance is noted, and much of the genetic variation occurs within samples and among populations, with little variation within each population. These results are, on some level, unsurprising for a species that is not only clonal but also includes minimal sexual reproduction. Sampling issues, such as small numbers of individuals studied for some populations and potential collection of ramets, could also have contributed to limited within-population genetic diversity. Additionally, almost all populations have negative *F*_IS_ values, a finding frequently occurring with clonal plants [17]. A similar result was recovered by Edgeloe et al. [18] for another clonal, polyploid species, *Posidonia australis* Hook.f. Despite the clonal growth in these polyploid species, the multiple gene copies may provide sufficient genetic diversity and potential so that rare species, such as *I. lacustris*, do not suffer the negative long-term impacts of vegetative reproduction and inbreeding. The changing climate will certainly be a test as to whether the genetic diversity harbored in each population will be appropriate to adapt to new conditions [19].

Among the identified clusters of populations, there are two notable areas: Bois Blanc Island in the eastern part of the sampled range and the four western populations. In Bois Blanc Island, the populations display mixed ancestry between the eastern and central populations, and these were results recovered with multiple datasets and analyses. This mixed ancestry could occur because of hybridization on the island itself with ancestors from both populations colonizing and interbreeding there. Alternatively, hybridization could have taken place on the mainland of the lower peninsula of Michigan, such as at MI7 or MI8, followed by colonization of the island. While the signature of mixed ancestry identified in the present study may suggest that hybridization is recent, given that the species reproduces clonally, the signature of (older) hybridization could remain for an extended period of time. It is useful to keep in mind that the island and nearby areas on the mainland are some of the more heavily sampled geographic regions in the present study. This greater sampling could hint at a similar pattern in other areas if individuals were sampled to a larger extent. It was not possible to include representatives from Ontario, Canada in the study, and future studies that add these will likely have greater context for the relationship of the central and eastern populations to those even farther east.

The four populations in the western cluster (MI6, MI16, WI4, and WI5) are notable. While these populations form a cluster in most analyses (Figure 2), the two Wisconsin populations (WI4 and 5) differ from those in Michigan, and, in some analyses, from each other. While WI4 and WI5 are geographically close together on the Door Peninsula and tend to cluster together in some analyses, WI4 is sometimes resolved as sharing ancestry with the eastern populations, which is not the case for WI5. This could be due to the retention of ancestral polymorphism or the fact that the establishment of each of these populations differs. However, in analyses that account for both genetic and geographic data (i.e., tess3r and conStruct), both Wisconsin populations are distinct clusters and/or are usually allied with the other western populations. This is particularly the case for the diploid dataset. In another, well-known Great Lakes shoreline endemic, *Cirsium pitcheri* Torr. & A.Gray, a similar pattern was recovered. The populations from the Door Peninsula are also quite distinct from others on Lake Michigan [20], and the northern populations on the peninsula share more alleles with the populations in the Upper Peninsula of Michigan than with some of the populations on the southern part of the peninsula.

MI6 and MI16 are intriguing populations of *I. lacustris* because they are situated inland, and this is not the case for the other sampled populations. While other populations can be found a short distance from the shoreline, these populations are ca. 30 km from the current boundary of Lake Michigan. These two populations are consistently recognized as genetically distinct from the other sampled populations, and these both likely became established during higher water level periods of Glacial Lake Algonquin ca. 12,500 years ago [21,22]. As water levels decreased during the time of Glacial Lake Chippewa and subsequently rose to current levels, these two populations became isolated in suitable habitat (e.g., conifer wetland) that allowed individuals of *I. lacustris* to persist, but without the opportunity to interbreed with other, coastal populations, resulting in their distinct genetic signature (Figure 2).

### 3.2. Migration and Demography

After deglaciation, *I. lacustris* migrated eastward from the western part of its range. This pattern provides evidence that MI6 and MI16 became established early in the colonization of the species during times of higher water levels and, therefore, are relicts rather than the result of inland dispersal. Additionally, the central and then eastern populations developed via migration across northern Lakes Michigan and Huron, and these populations may have retained some of the ancestral polymorphisms in the more western populations, such as WI4 and WI5. This west-to-east pattern suggests that the populations in Ontario are the most recently established, a hypothesis that can be tested during a future study. The pattern noted here for *I. lacustris* differs from that of *C. pitcheri*, which is hypothesized to have migrated from east to west [20].

Overall, rates of migration, as inferred with BA3-SNPs, among populations are minimal, a result recovered in other species of *Iris* on the Korean Peninsula [23] and a pattern that is not uncommon for narrow endemics [20]. This minimal migration is the case for all 24 populations studied as well as with three and four population clusters inferred (Figure 3). Although the species presently reproduces within populations, migration occurred and may have provided an infusion of new alleles, even if this was not a common occurrence.

In *C. pitcheri*, Fant et al. [20] note that the changes in the water level of the Great Lakes shaped the geographic distribution of this endemic species, with lower water levels allowing for increased connection among populations. Lake level changes could also have impacted the geographic distribution of *I. lacustris*. This is particularly the case for the more inland populations, which could have become established ca. 4500 years ago during the most recent high water levels for the lake. Lower lake levels may have influenced colonization of the islands as well as migration across the northern regions of Lake Michigan and allowed for the exchange of individuals that currently would be more challenging.

An alternative hypothesis for the present geographic distribution of the species also exists. Van Kley and Wujek [6] and Brotske [4] provide evidence that *I. lacustris* can inhabit a diversity of ecosystems and that changes in patterns of disturbance and forest succession following European colonization of the area reduced the suitable habitat for the species (e.g., more forests with more closed canopies). This has resulted in populations primarily being restricted to shorelines where habitat was appropriate. If this is the case, the inland populations, such as MI6, would still represent relicts of a prior time, but this would be due to remnant habitat availability based on adequate disturbance regimes and/or seral stages, not prior establishment during higher water levels of the Great Lakes and subsequent serendipitous survival.

### 3.3. Subsetting Diploid and Tetraploid Loci

In the present study, polyRAD [24] was used to create datasets of diploid and tetraploid loci, and these were analyzed alongside a dataset of all loci for the MCR90 and MCR50 datasets. In general, analyses of all six datasets produced fairly similar results (Figure 2, Table 3 and Table 4). fastSTRUCTURE analyses of MCR90 and MCR50 datasets of all loci resulted in the identification of a cluster of six populations in the central part of the sampled population of *I. lacustris* (MI2, MI3, MI4, MI11, MI12, and MI20) that was not recovered with the diploid or tetraploid datasets, although hints of this cluster can be seen in the MCR90 2N dataset at K = 5. This cluster is identified in all of the datasets with loci under selection as either one or two clusters (Figure 2) and with the MCR90 datasets analyzed with STRUCTURE [25] and MavericK [26].

The similar results among the datasets, regardless of ploidy, may provide some evidence that not disentangling diploid and tetraploid loci from all loci may not lead to spurious results using SNP data for population genomics [27]. This statement should be treated with skepticism because it is based only on one, empirical, study. Others who have used polyRAD to subset their datasets and identify diploid loci to use for population genomics [28,29], which is a practice aligned with assumptions of common methods [28], have not explored the use of all loci and/or tetraploid loci in comparison to only ones that segregate as diploids. It would be useful for additional studies on the population genomics of polyploid species to examine data employing all, diploid, and tetraploid (and higher) loci to determine if similar or divergent results are recovered. At the same time, the results presented herein may provide some level of confidence for researchers investigating the population genomics of species of unknown ploidy that use all loci identified via tGBS, and similar reduced-representation methods may not yield incongruent results.

### 3.4. Conservation Genetics of I. lacustris

The evolutionarily significant units (ESUs) were described above with all loci used for population genomic analyses, and the management units (MUs), which were determined using only loci not under selection, are similar, but not identical to the ESUs; however, the differences are minor (Figure 4). Given the similar ESUs and MUs, the management of the populations of *I. lacustris* could be geographically clustered into three to four units. However, the results of the use of the loci under selection to resolve adaptive units (AUs) differ from those of ESUs and MUs (Supplemental Figure S18). The AUs provide evidence of local adaptation, so managing only three or four MUs would not necessarily ensure that all of the genetic diversity of the species is appropriately protected. A total of nine AUs are recognized (Table 1), and while these are also geographically clustered, the AUs are much smaller than are the ESUs and MUs (Figure 4).

This local adaptation is, on some level, unsurprising, because even though the species is generally restricted to the same type of habitat presently (i.e., shorelines), climatic, soil, and vegetation differences occur across the geographic range of the species. Indeed, *I. lacustris* inhabits three of the landscape ecology regions of Michigan and multiple districts and subdistricts within each region [30,31]. Van Kley and Wujek [6] also recognized four soil types, four vegetation types, and pH variation across the species’ range. Given that the species primarily reproduces asexually, this can lead to a loss of genetic variation over time as a limited number of successful genotypes dominates each particular climate–soil–vegetation combination. Consequently, the seemingly same type of habitat in a geographically distinct area may result in local adaptations to the specific region and ecosystem and contribute to outbreeding depression, limiting successful offspring from infrequent interpopulation crosses.

## 4. Materials and Methods

### 4.1. Plant Material

During the summers of 2019 and 2020, leaf material of 171 individuals of *I. lacustris* was collected from 24 locations in Michigan and Wisconsin (Figure 1) and dried in silica gel. The number of individuals per population ranged from 1 to 12, depending on the suitability of the population for collection. Most individual plants were collected at least 3 m from each other to maximize the possibility of sampling genets, not ramets. Latitude and longitude were recorded for each specimen.

### 4.2. DNA Sequencing

Leaf material was sent to data2bio (www.data2bio.com, accessed on 1 May 2023) for DNA isolation and tunable Genotyping-by-Sequencing (tGBS) to recognize single nucleotide polymorphisms (SNPs) across the populations. Using the restriction enzyme Bsp1286I, paired-end tGBS libraries were created [15] and subsequently sequenced with an Illumina HiSeq X (Illumina Inc., San Diego, CA, USA). Based on all sequence data, consensus reference sequences were generated with CD-HIT-454 [32] after sequencing depth was normalized to 50×, and sequencing errors were corrected using Fiona [33]. Low-quality reads were discarded (PHRED quality < 15 and error rates ≥ 3%) and trimmed, and GSNAP [34] was employed to map reads to the reference sequences based on the following parameters: ≤2 mismatches per 36 bp and less than five total per 75 bp for tails. SNPs were identified based on the following criteria: two most common alleles supported by at least 30% of the aligned bases, at least five unique reads, the sum of the one or two most common alleles covering at least 80% of the aligned reads, and no polymorphisms in the first or last three base pairs of each read. From the SNPs, two datasets were created: MCR90 with up to 10% missing data and MCR50 with up to 50% missing data.

### 4.3. Polyploidy Filtering

Because I. lacustris is a putative polyploid and many population genetic methods assume that species are (at most) diploid, polyRAD [24] was used to identify and filter loci that are diploid and tetraploid. The MCR90 and MCR50 datasets were filtered using the IteratePopStruct command to identify genotypes, and then the H_ind_/H_E_ statistic [24,35] was employed to recognize diploid loci with H_ind_/H_E_ < 0.5 and tetraploid loci with H_ind_/H_E_ > 0.75. Datasets were created for each set of loci (Table 2). The number of SNPs in the diploid and tetraploid datasets does not equal the value in the initial datasets because of filtering with polyRAD.

### 4.4. Population Genomics

Observed and expected heterozygosity measurements and *F*-statistics were calculated with hierfstat [36,37], and AMOVA was conducted with poppr [38]. All 24 populations were examined, as were the populations divided into three and four geographic clusters, which are based on the optimal K values from preliminary analyses in fastSTRUCTURE (Table 2) and patterns of population structure from STRUCTURE and MavericK. fastSTRUCTURE [39] was employed to identify population structure, including the optimal number of clusters (K), and for these analyses, K = 1–24 were analyzed for the six SNP datasets, using Structure_threader [40], on the Kettering University High-Performance Computing Cluster (KUHPC). Ten replicates were run for each K, with a convergence criterion of 0.000001, a simple prior, and 100 test sets for cross-validation. The CLUMPAK main pipeline, which includes CLUMPP [41] and DISTRUCT [42], was employed to organize, cluster, and visualize the results of independent fastSTRUCTURE analyses, via 10,000 permutations of the LargeKGreedy algorithm [43]. To identify the optimal K value(s), the marginal likelihood that maximizes model complexity from fastSTRUCTURE and the MedMedK, MedMeanK, MaxMedK, and MaxMeanK values determined by StructureSelector [44,45] were examined. These latter four metrics are useful for uneven sampling and are based on recognizing the number of clusters that include, at minimum, one subpopulation. Differences among these metrics are the result of the arithmetic mean or median used and the median or maximum number of clusters identified [45].

For comparison, and given potential variation in ploidy at loci [27], STRUCTURE [25] and MavericK [26] were also used, with Structure_threader, for analyses with the three MCR90 datasets. With STRUCTURE, the following parameters were used with K = 1–24: 1,000,000 steps and 500,000 burnin, with alpha and lambda of 1, and with or without admixture. Ten replicates were run for each K. CLUMPAK and StructureSelector were also used for STRUCTURE analyses, with the best K also determined via the method of Evanno et al. [46] and Ln Pr (X|K). MavericK analyses were run for K = 1–12 with five replicates per K, without admixture, using the following parameters for each replicate: 50,000 steps and 5000 burnin for Markov Chain Monte Carlo (MCMC) sampling and an alpha of 1500 steps and 5000 burnin, with 50 rungs, for thermodynamic integration (TI) sampling, and 100 expectation-maximization repeats. With MavericK, graphs were visualized with R [47], and the optimal K value was determined using TI.

To explicitly include geographical data along with SNPs to investigate patterns of population genetics, tess3r [48] and conStruct [49] were used, and all datasets were analyzed with the former, but only the three MCR90 datasets with the latter. For tess3r, the alternating projected least squares method was undertaken for K = 1–24 for MCR90 and K = 1–12 for MCR50 datasets. Results for each K were visualized with bar graphs and maps in R [47], and the optimal K value was identified using the cross-validation plot for each dataset. For conStruct cross-validation, analyses were conducted with five replicates, for K = 1–8, using 10,000 MCMC iterations sampled every 1000 iterations and a training proportion of 0.5–0.8, depending on the dataset. Subsequently, analyses with K = 3–5 were conducted, with five replicates, using one chain run for 100,000 MCMC iterations sampled every 1000 iterations and with the spatial model.

In addition to analyses for explicit population structure, all datasets were analyzed with principal component analyses (PCA), correspondence analyses (CA), and discriminant analyses of principal components (DAPC) in adegenet [50], principal coordinate analyses (PCoA) in hierfstat [36,37], and isolation-by-distance (IBD) analyses in adegenet using separate Mantel tests for population and individuals, with 999 simulations for the Mantel test. For DAPC for each dataset, the Bayesian Information Criterion (BIC) was used to identify the optimal number of clusters, and cross-validation was employed to explore the most appropriate number of PCs to retain for analysis.

Loci under selection were determined with BayeScan [51] using 100,000 iterations, a burnin of 50,000 iterations, a thinning interval of 10, and a sample size of 5000, and for each analysis, 20 pilot runs were conducted, each with 5000 steps. Loci under selection were visualized in R using *F*_ST_ values and a false discovery rate of 0.05.

Demographic history and patterns of migration were explored using BA3-SNPs [52,53], DIYABC Random Forest (DIYABC-RF) [54], and abcranger [55], and only the three MCR90 datasets were used for these analyses, with the three and four aforementioned population clusters used (apart from all 24 populations investigated with MCR90 with BA3-SNPs). For BA3-SNPs, the datasets were each run for 50 million Markov Chain Monte Carlo (MCMC) iterations, with 20 million MCMC burnin iterations, and a sampling interval of 2500 iterations, and the initial parameters for allele frequencies, inbreeding coefficient, and migration rates were tuned to vary between 0.2–0.6. For DIYABC-RF, the optimal scenario for patterns of diversification were examined among all 15 arrangements of four bifurcating populations. For each scenario, population size was modelled to vary after populations split and one and two other times for when the second and first populations diverge (Supplemental Figure S17). For analyses, all genetic diversity, *F*_ST_ distances, Nei’s distances, and admixture estimates were selected, and the analyses were run for 15 million simulations with a batch size of 1000. Using the results of the training, a random forest analysis was conducted with abcranger [55] using 1000 trees to identify the number of trees supporting each model and to estimate the parameters of the model, with and without linear discriminant analysis, for partial least squares (PLS) estimation on the optimal model for each dataset.

### 4.5. Conservation Units

Conservation and management units were identified following the three-step method of Funk et al. [16], in which (1) evolutionarily significant units (ESUs) are recognized using all loci, (2) management units (MUs) are delimited with non-outlier loci, and (3) adaptive groups are determined using outlier loci. For the three steps, fastSTRUCTURE [39], PCA, and DAPC were used [50]. The first step was described above for datasets with all loci, and the other two steps were conducted using the same parameters for the three analyses and were based on two datasets (loci under and not under selection as determined via BayeScan [51]) for each MCR50 dataset and the all loci dataset of MCR90 (Table 2). The optimal K value was identified using StructureSelector [44], the marginal likelihood that maximizes model complexity from fastSTRUCTURE [39], and the BIC for DAPC with adegenet [50]. Based on the results of these analyses, ESUs, MUs, and adaptive groups were identified (Supplemental Figure S18).

## 5. Conclusions

The present study provides evidence of genomic variation and local adaptation across the geographic range of the species, which is novel given the negligible genetic diversity previously recovered for *I. lacustris* [8,9,10]. However, as Van Kley and Wujek [6] stated thirty years ago, “Despite a preference for a somewhat disturbed habitat, *Iris lacustris* will not grow where the habitat has been destroyed by residential, resort, or industrial development”. Therefore, the conservation genetic results are of limited value if management steps are not taken to ensure that individuals of *I. lacustris* have the opportunity to be successful in situ. This includes not only ensuring intermediate light conditions and limited litter [5,6], but also that as much genetic diversity across the entire geographic range of the species is conserved and managed appropriately. Indeed, given the local genetic diversity recognized among the nine adaptive units, it would be prudent to strive to conserve representatives from these areas. This is particularly important because the populations that are best able to adapt to the changing climate in the Great Lakes region is presently unknown [56]. Therefore, to ensure the longevity of this charismatic species, appropriate long-term management is necessary. Future work that includes the populations of *I. lacustris* from Ontario can extend the presented results to investigate the ways in which these populations relate to those in the United States. Given the international geographic range of the species, conservation efforts that are binational would be particularly useful.

## Figures and Tables

**Table 1:** Population and sampling information and assignation of populations to clusters based on results of various population genomic analyses, including recognition of management and adaptive units, based on loci not under and under selection, respectively. Cluster, management unit, and adaptive unit assignation is based on population genetic analyses with fastStructure, discriminant analysis of principal components (DAPC), principal component analyses (PCA), and others described in the text.

Populations Sampled	Number of Individuals Sampled	Four Population Clusters in Analyses	Three Population Clusters in Analyses	Management Units (All Loci)	Management Units (Diploid and Tetraploid Loci)	Adaptive Units
MI1	10	East	East	1	1	1
MI2	3	Mid1	Mid	2	2	2
MI3	8	Mid1	Mid	2	2	3
MI4	7	Mid1	Mid	2	2	3
MI5	7	Mid2	Mid	3	3	4
MI6	14	West	West	4	4	5
MI7	8	Mid2	Mid	2	2	4
MI8	4	Mid2	Mid	3	3	4
MI9	3	Mid2	Mid	2	2	6
MI10	5	East	East	1	3	7
MI11	1	Mid1	Mid	2	2	3
MI12	2	Mid1	Mid	2	2	3
MI13	3	Mid2	Mid	3	3	4
MI14	13	East	East	1	3	7
MI15	8	East	East	1	1	7
MI16	3	West	West	4	2	5
MI17	10	Mid2	Mid	2	2	6
MI18	10	East	East	1	1	1
MI19	10	Mid2	Mid	2	2	6
MI20	3	Mid1	Mid	2	2	2
MI21	7	East	East	1	3	1
MI22	8	Mid2	Mid	3	3	4
WI4	12	West	West	4	2	8
WI5	12	West	West	4	2	9

**Table 2:** Information on six SNP (single nucleotide polymorphism) datasets examined including best K (cluster) value under various analyses. Dashes indicate analysis was not performed for dataset. StructureSelector results include MedMedK, MedMeanK, MaxMedK, and MaxMeanK, and, therefore, may have a range of best K values due to different results from these four metrics. DAPC is discriminant analysis of principal components, and for these analyses, best K value is determined via Bayesian Information Criterion. Additional information on identification of loci under selection and best K values in text.

			All Loci		Loci under Selection		Loci Not under Selection	
Dataset	SNPs	Loci under Selection	StructureSelector	DAPC	StructureSelector	DAPC	StructureSelector	DAPC
MCR90	5354	401	6	9	12–14	13	3–4	7
MCR90 diploid loci	2106	29	4–5	7	-	-	-	-
MCR90 tetraploid loci	1382	21	4–5	6	-	-	-	-
MCR50	344,509	65,075	5–7	4	11–13	10	3	1
MCR50 diploid loci	50,134	4311	3–4	2–3	9–10	7	2–3	1
MCR50 tetraploid loci	82,237	6939	3–4	2–3	8	9	3	1

**Table 3:** Observed, expected, and total heterozygosity (H_O_, H_S_, H_T_) and fixation index (F_IS_) for the three MCR90 datasets for each population. Sample sizes are less than five for MI2, MI8, MI9, MI11, MI12, MI13, MI16, MI20, which could impact calculated statistics.

	MCR90 All Loci				MCR90 Diploid Loci				MCR90 Tetraploid Loci			
Population	H_O_	H_S_	H_T_	F_IS_	H_O_	H_S_	H_T_	F_IS_	H_O_	H_S_	H_T_	F_IS_
MI1	0.0586	0.0516	0.0516	−0.1365	0.064	0.0519	0.0519	−0.2325	0.0548	0.0462	0.0462	−0.1875
MI2	0.0503	0.0411	0.0411	−0.2224	0.0538	0.0417	0.0417	−0.2883	0.0532	0.0431	0.0431	−0.2329
MI3	0.0472	0.0307	0.0307	−0.5394	0.0521	0.0322	0.0322	−0.6191	0.0474	0.0301	0.0301	−0.5768
MI4	0.0581	0.0451	0.0451	−0.2873	0.0651	0.0479	0.0479	−0.3582	0.0628	0.0497	0.0497	−0.2624
MI5	0.0558	0.0532	0.0532	−0.047	0.052	0.0428	0.0428	−0.2161	0.051	0.041	0.041	−0.2444
MI6	0.0957	0.0704	0.0704	−0.3593	0.1043	0.0742	0.0742	−0.4064	0.0933	0.0675	0.0675	−0.3826
MI7	0.054	0.049	0.049	−0.1021	0.0519	0.042	0.042	−0.2372	0.0559	0.0449	0.0449	−0.2455
MI8	0.0655	0.0563	0.0563	−0.1631	0.0677	0.0573	0.0573	−0.1816	0.067	0.0555	0.0555	−0.2074
MI9	0.0594	0.0401	0.0401	−0.4814	0.0586	0.0373	0.0373	−0.5714	0.0673	0.0442	0.0442	−0.5217
MI10	0.0612	0.0477	0.0477	−0.2842	0.0554	0.043	0.043	−0.289	0.0515	0.0383	0.0383	−0.3455
MI11	0.0475	-	-	-	0.0527	-	-	-	0.0499	-	-	-
MI12	0.0522	0.0385	0.0385	−0.3578	0.0592	0.0411	0.0411	−0.4413	0.0551	0.0433	0.0433	−0.2749
MI13	0.0557	0.0447	0.0447	−0.2463	0.0508	0.0409	0.0409	−0.2434	0.0543	0.0401	0.0401	−0.3551
MI14	0.0535	0.0488	0.0488	−0.0981	0.0542	0.0448	0.0448	−0.2105	0.0497	0.0415	0.0415	−0.1989
MI15	0.0573	0.0551	0.0551	−0.0395	0.059	0.0516	0.0516	−0.1434	0.0589	0.0502	0.0502	−0.1724
MI16	0.0961	0.0694	0.0694	−0.384	0.0956	0.0661	0.0661	−0.4464	0.0795	0.0541	0.0541	−0.4686
MI17	0.0671	0.062	0.062	−0.0827	0.0609	0.0488	0.0488	−0.2478	0.0647	0.0524	0.0524	−0.2357
MI18	0.0639	0.0567	0.0567	−0.1277	0.0672	0.0542	0.0542	−0.2401	0.0643	0.0534	0.0534	−0.2054
MI19	0.0651	0.0575	0.0575	−0.1324	0.0645	0.0512	0.0512	−0.2604	0.0591	0.0487	0.0487	−0.215
MI20	0.0467	0.0343	0.0343	−0.3597	0.0481	0.0351	0.0351	−0.3711	0.0516	0.0365	0.0365	−0.4123
MI21	0.0624	0.054	0.054	−0.1557	0.0628	0.0497	0.0497	−0.262	0.0637	0.0512	0.0512	−0.2435
MI22	0.0543	0.0492	0.0492	−0.1035	0.0464	0.0382	0.0382	−0.2135	0.049	0.0397	0.0397	−0.2343
WI4	0.1081	0.0946	0.0946	−0.1424	0.1032	0.0848	0.0848	−0.2179	0.0895	0.0745	0.0745	−0.201
WI5	0.1033	0.085	0.085	−0.2157	0.1015	0.0775	0.0775	−0.3102	0.094	0.0734	0.0734	−0.2814

**Table 4:** Pairwise F_ST_ values and heatmap for MCR90 all loci (below diagonal) and MCR90 diploid loci (above diagonal). Below the diagonal, red indicates lower values, and blue is for higher values. Above the diagonal, yellow is for lower values, and green is for higher values. Sample sizes are less than five for MI2, MI8, MI9, MI11, MI12, MI13, MI16, MI20, which could impact calculated statistics.

	MI1	MI2	MI3	MI4	MI5	MI6	MI7	MI8	MI9	MI10	MI11	MI12	MI13	MI14	MI15	MI16	MI17	MI18	MI19	MI20	MI21	MI22	WI4	WI5
MI1	-	0.17	0.20	0.21	0.16	0.25	0.18	0.16	0.22	0.14	0.12	0.17	0.13	0.11	0.09	0.26	0.18	0.05	0.20	0.16	0.05	0.15	0.15	0.19
MI2	0.29	-	0.12	0.12	0.16	0.24	0.16	0.18	0.24	0.20	0.07	0.11	0.13	0.16	0.15	0.27	0.16	0.19	0.17	0.00	0.19	0.11	0.16	0.20
MI3	0.36	0.26	-	−0.01	0.19	0.21	0.19	0.20	0.26	0.27	0.00	0.05	0.22	0.21	0.20	0.28	0.13	0.22	0.14	0.14	0.22	0.18	0.13	0.16
MI4	0.37	0.26	0.01	-	0.19	0.24	0.18	0.19	0.20	0.25	−0.08	0.03	0.19	0.22	0.20	0.26	0.14	0.23	0.15	0.10	0.23	0.18	0.16	0.19
MI5	0.26	0.29	0.30	0.31	-	0.20	0.08	0.04	0.20	0.12	0.12	0.18	0.08	0.12	0.12	0.23	0.12	0.17	0.14	0.16	0.16	0.06	0.14	0.15
MI6	0.41	0.39	0.37	0.39	0.31	-	0.23	0.19	0.22	0.24	0.14	0.19	0.20	0.24	0.23	0.13	0.20	0.25	0.20	0.21	0.24	0.22	0.19	0.20
MI7	0.32	0.35	0.37	0.37	0.12	0.35	-	0.08	0.19	0.19	0.11	0.18	0.16	0.15	0.15	0.25	0.12	0.20	0.14	0.14	0.18	0.11	0.15	0.18
MI8	0.31	0.38	0.41	0.40	0.07	0.34	0.14	-	0.18	0.15	0.04	0.14	0.10	0.14	0.12	0.20	0.11	0.17	0.13	0.16	0.16	0.09	0.12	0.15
MI9	0.42	0.43	0.44	0.38	0.32	0.40	0.34	0.39	-	0.29	0.18	0.25	0.28	0.24	0.21	0.25	0.10	0.24	0.12	0.25	0.25	0.24	0.13	0.14
MI10	0.20	0.34	0.44	0.42	0.23	0.38	0.32	0.29	0.45	-	0.19	0.25	0.13	0.03	0.06	0.28	0.20	0.13	0.21	0.21	0.09	0.12	0.14	0.19
MI11	0.30	0.24	0.05	−0.07	0.21	0.31	0.29	0.30	0.38	0.38	-	−0.03	0.12	0.14	0.10	0.13	0.05	0.14	0.06	0.07	0.14	0.12	0.02	0.07
MI12	0.33	0.25	0.07	0.03	0.26	0.35	0.34	0.36	0.41	0.41	0.01	-	0.17	0.19	0.15	0.21	0.13	0.18	0.13	0.14	0.20	0.18	0.09	0.13
MI13	0.21	0.26	0.37	0.33	0.16	0.33	0.27	0.23	0.44	0.22	0.28	0.31	-	0.09	0.09	0.25	0.15	0.14	0.16	0.15	0.14	0.02	0.12	0.15
MI14	0.15	0.31	0.38	0.39	0.24	0.40	0.30	0.28	0.42	0.04	0.33	0.36	0.18	-	0.03	0.26	0.18	0.10	0.19	0.15	0.07	0.09	0.14	0.19
MI15	0.17	0.32	0.38	0.39	0.24	0.39	0.29	0.26	0.41	0.09	0.30	0.34	0.19	0.04	-	0.23	0.16	0.09	0.18	0.13	0.08	0.11	0.12	0.17
MI16	0.43	0.44	0.44	0.43	0.32	0.19	0.37	0.34	0.42	0.43	0.30	0.37	0.39	0.42	0.39	-	0.20	0.25	0.20	0.26	0.26	0.27	0.14	0.14
MI17	0.28	0.25	0.22	0.25	0.17	0.32	0.21	0.22	0.18	0.27	0.13	0.20	0.21	0.27	0.26	0.28	-	0.20	0.07	0.14	0.18	0.13	0.13	0.16
MI18	0.12	0.33	0.38	0.40	0.29	0.41	0.33	0.32	0.42	0.17	0.32	0.35	0.23	0.14	0.15	0.41	0.29	-	0.21	0.18	0.04	0.16	0.15	0.21
MI19	0.33	0.30	0.28	0.31	0.22	0.35	0.25	0.25	0.27	0.32	0.21	0.26	0.26	0.31	0.30	0.34	0.11	0.33	-	0.15	0.20	0.15	0.15	0.17
MI20	0.29	−0.01	0.29	0.25	0.27	0.36	0.33	0.38	0.45	0.35	0.24	0.28	0.29	0.31	0.31	0.42	0.22	0.32	0.28	-	0.18	0.10	0.12	0.16
MI21	0.09	0.34	0.39	0.40	0.26	0.39	0.31	0.30	0.42	0.13	0.32	0.36	0.21	0.09	0.13	0.41	0.27	0.05	0.32	0.33	-	0.14	0.14	0.19
MI22	0.19	0.22	0.28	0.30	0.09	0.33	0.17	0.15	0.34	0.17	0.21	0.26	0.04	0.15	0.17	0.36	0.16	0.22	0.21	0.21	0.18	-	0.15	0.18
WI4	0.27	0.26	0.23	0.27	0.22	0.30	0.28	0.24	0.27	0.22	0.12	0.19	0.20	0.24	0.23	0.25	0.20	0.25	0.25	0.21	0.24	0.22	-	0.15
WI5	0.33	0.32	0.24	0.28	0.26	0.27	0.30	0.27	0.27	0.30	0.16	0.21	0.26	0.32	0.30	0.21	0.23	0.33	0.27	0.28	0.31	0.26	0.24	-

## Data Availability

Data files in VCF format are available at the Dryad repository (https://doi.org/10.5061/dryad.xwdbrv1jh).

## Supplementary Materials

The following supporting information can be downloaded at: https://www.mdpi.com/article/10.3390/plants12132557/s1, Figure S1. Results for Isolation-by-Distance (IBD) for the six datasets. The x-axis is geographic distance, and the y-axis is genetic distance. Figure S2. Structure bar graphs from STRUCTURE for the six datasets analyzed in the present study for K (clusters) = 3–5. Individual ancestry denoted by color. Populations are denoted below each graph. Figure S3. Structure bar graphs from MavericK, without admixture, for the three MCR90 datasets analyzed in the present study for K (clusters) = 3–5. Individual ancestry denoted by color. Populations are denoted below each graph. Figure S4. tess3r maps of population assignation for the six datasets analyzed in the present study for K (clusters) = 3–5. Individual ancestry denoted by color. Figure S5. Maps and bar graphs of population assignation for the three MCR90 datasets analyzed in the present study for K (clusters) = 3–5. Individual ancestry denoted by color. Figure S6. Results for best K from StructureSelector for analyses with fastStructure for (A) MCR90 all loci, (B) MCR90 diploid loci, and (C) MCR90 tetraploid loci. Figure S7. Results for best K from StructureSelector for analyses with fastStructure for (A) MCR50 all loci, (B) MCR50 diploid loci, and (C) MCR50 tetraploid loci. Figure S8. Results for best K from StructureSelector for analyses with Structure without admixture for (A) MCR90 all loci, (B) MCR90 diploid loci, and (C) MCR90 tetraploid loci. Figure S9. Results for best K from StructureSelector for analyses with Structure with admixture for (A) MCR90 all loci, (B) MCR90 diploid loci, and (C) MCR90 tetraploid loci. Figure S10. Results for best K from MavericK, based on thermodynamic integration (TI), for analyses without admixture (A) MCR90 all loci, (B) MCR90 diploid loci, and (C) MCR90 tetraploid loci. Figure S11. Results for cross-validation scores for tess3r analyses for (A) MCR90 all loci, (B) MCR90 diploid loci, (C) MCR90 tetraploid loci, (D), MCR50 all loci, (E) MCR50 diploid loci, and (F) MCR50 tetraploid loci. Figure S12. Results for cross-validation scores for conStruct validation analyses for (A) MCR90 all loci, (B) MCR90 diploid loci, and (C) MCR90 tetraploid loci to identify best K (clusters). Graphs with blue and green dots are for spatial and non-spatial models, respectively, and graph with only blue dots displays predictive accuracy for spatial model with confidence intervals. Figure S13. Results for Bayesian Information Criterion (BIC), to identify best K (clusters), from discriminant analysis of principal components (DAPC) for (A) MCR90 all loci, (B) MCR90 diploid loci, (C) MCR90 tetraploid loci, (D), MCR50 all loci, (E) MCR50 diploid loci, and (F) MCR50 tetraploid loci. Figure S14. Results from StructureSelector for best K (clusters) fastStructure analyses for loci under selection for (A) MCR90 all loci, (B) MCR50 all loci, (C) MCR50 diploid loci, and (D) MCR50 tetraploid loci. Figure S15. Results from StructureSelector for best K (clusters) fastStructure analyses for loci not under selection for (A) MCR90 all loci, (B) MCR50 all loci, (C) MCR50 diploid loci, and (D) MCR50 tetraploid loci. Figure S16. Results for Bayesian Information Criterion (BIC), to identify best K (clusters), from discriminant analysis of principal components (DAPC) analyses for (A) MCR90 all loci under selection, (B) MCR50 all loci under selection, (C) MCR50 diploid loci under selection, (D), MCR50 tetraploid loci under selection, (E) MCR90 all loci not under selection, (F) MCR50 all loci not under selection, (G) MCR50 diploid loci not under selection, (H), MCR50 tetraploid loci not under selection. Figure S17. 15 branching scenarios evaluated in DIYABC. Pop 1 is East, Pop 2 is Mid 1, Pop 3 is Mid 2, Pop 4 is West. See Table 1 for population assignation to each population. Change in color represents potential change in population size. Scenario 3 is optimal for all and tetraploid loci, and scenario 7 is optimal for diploid loci. Figure S18. Nine Adaptive Units recognized from population genetic analyses using loci under selection. Map of locations sampled in present study. Dark gray entire lines denote division between East, Mid1, Mid2, and West clusters (also recognized as Management Units). The dashed gray line separates Mid1 and Mid2 populations, and Mid includes both groups of populations together. Light gray lines separate Wisconsin (USA), Michigan (USA), and Ontario (Canada). Scale bar, in red, represents 50 kilometers. Table S1. AMOVA results for all datasets. Table S2. K values for the MCR90 datasets for STRUCTURE and Maverick.
