# Supplementary material for: The Conservation Genetics of Iris lacustris (Dwarf Lake Iris), a Great Lakes Endemic
Source: Plants (Basel). 2023 Jul 5;12(13):2557. doi: 10.3390/plants12132557 (PMC10346457; doi:10.3390/plants12132557)

Figure S1. Results for Isolation-by-Distance (IBD) for the six datasets. The x-axis is geographic distance, and the y-axis is genetic distance.

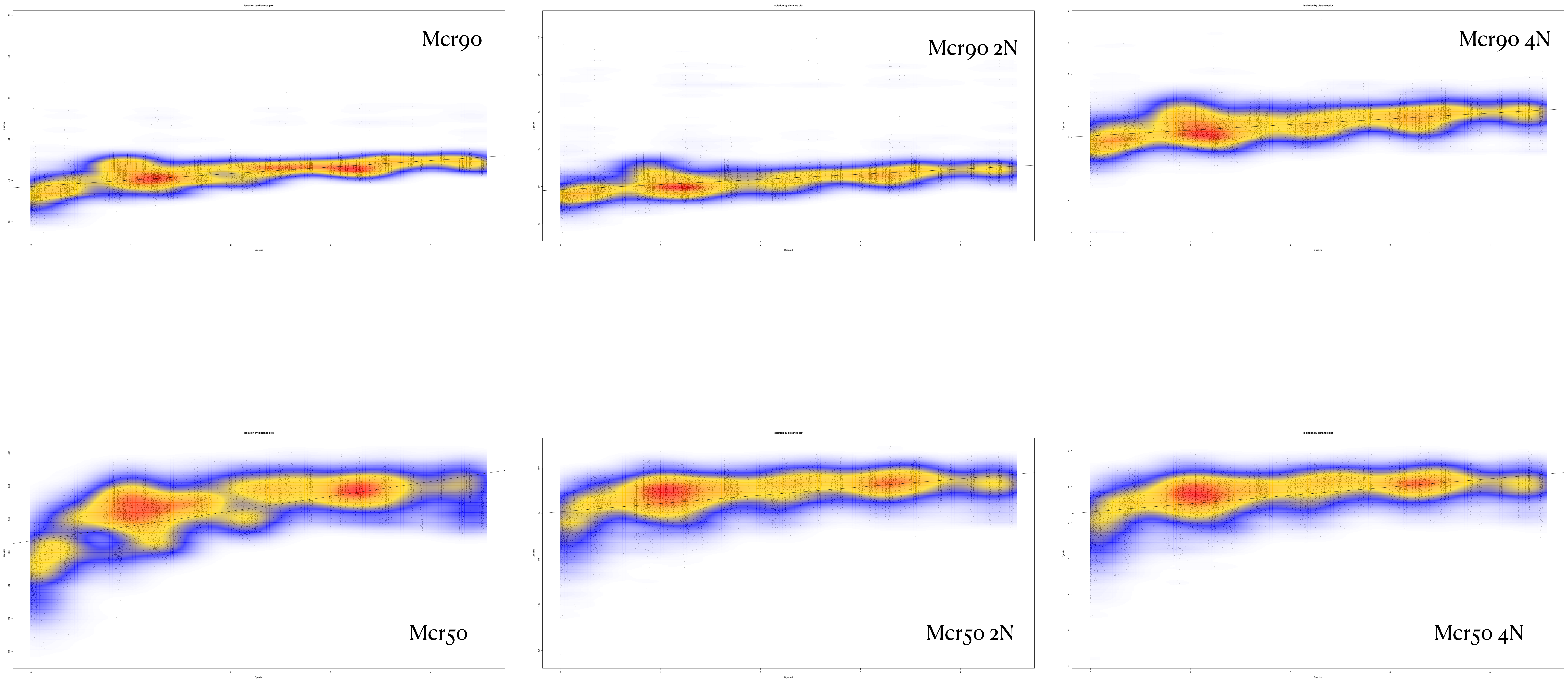

Figure S2. Structure bar graphs from STRUCTURE for the six datasets analyzed in the present study for K (clusters) = 3-5. Individual ancestry denoted by color. Populations are denoted below each graph.

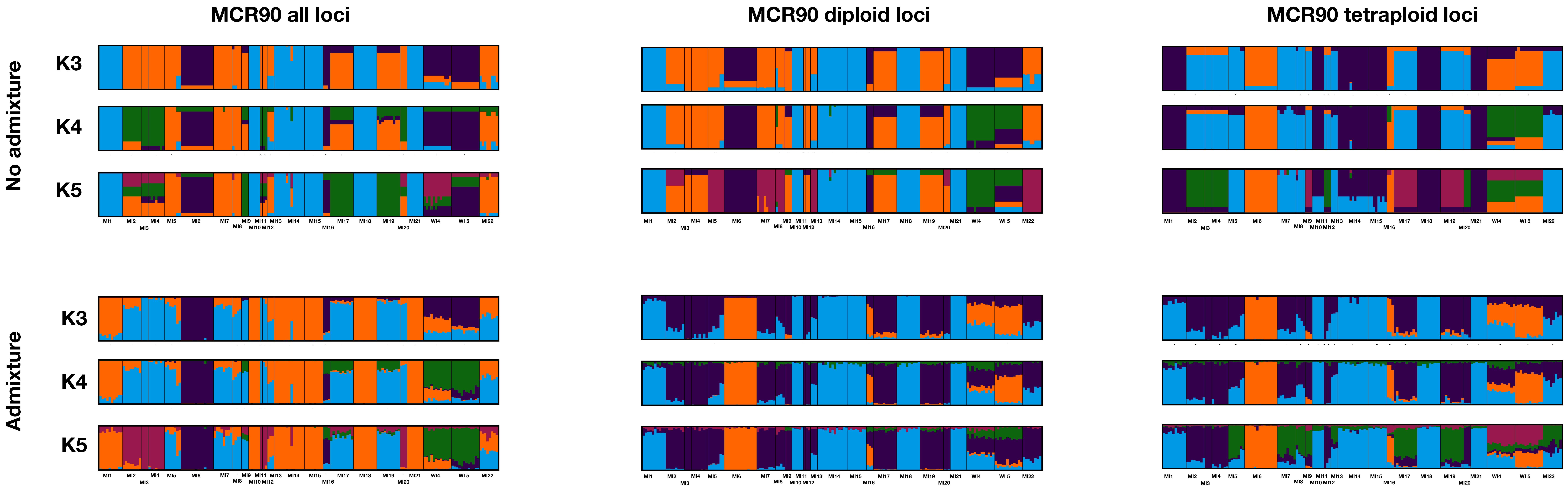

Figure S3. Structure bar graphs from MavericK, without admixture, for the three MCR90 datasets analyzed in the present study for K (clusters) = 3-5. Individual ancestry denoted by color. Populations are denoted below each graph.

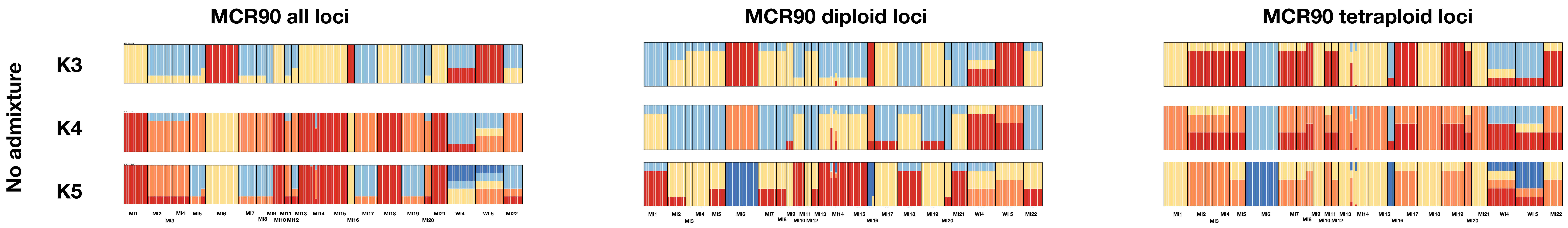

Figure S4. tess3r maps of population assignation for the six datasets analyzed in the present study for K (clusters) = 3-5. Individual ancestry denoted by color.

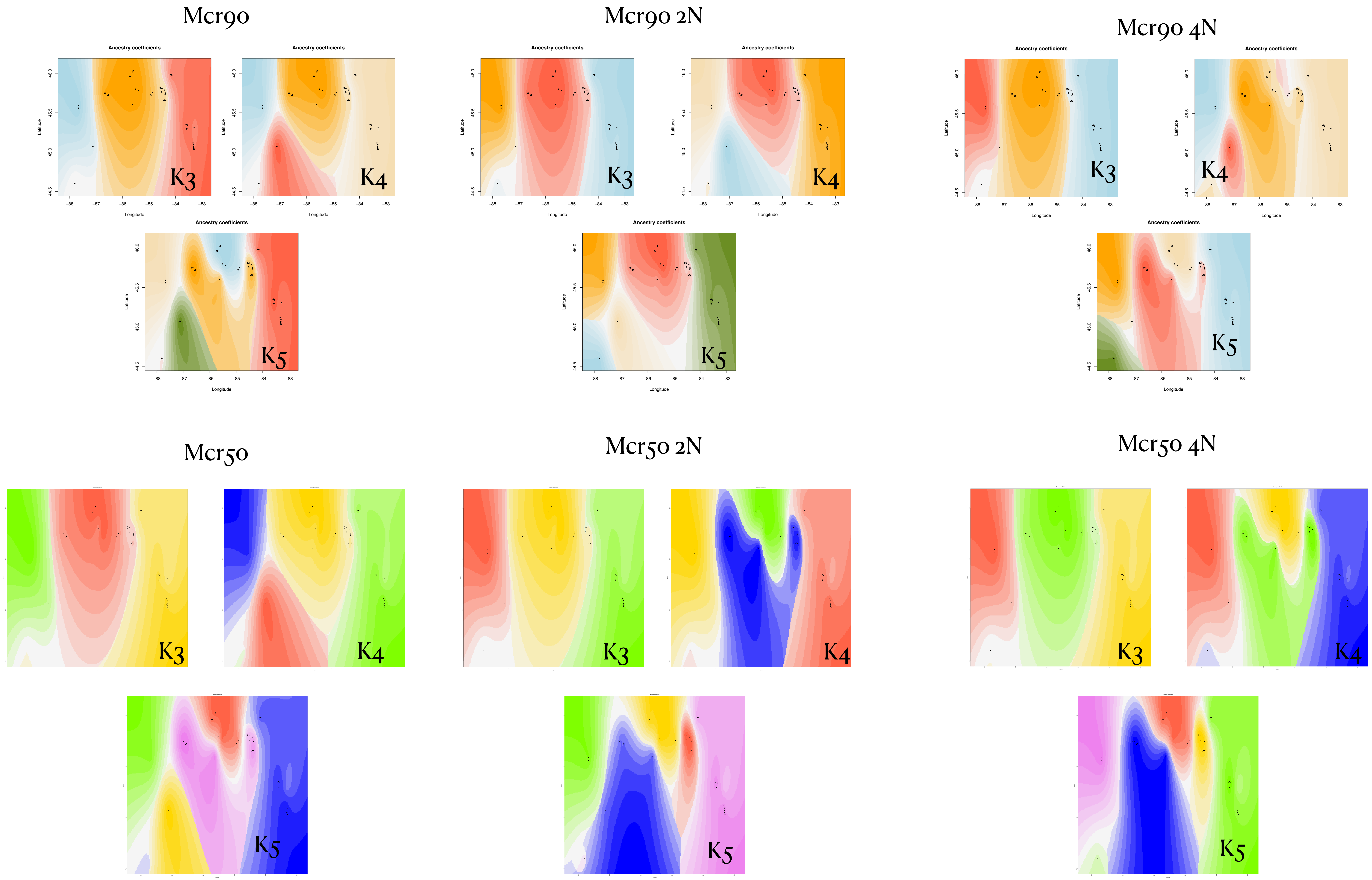

Figure S5. Maps and bar graphs of population assignation for the three MCR90 datasets analyzed in the present study for K (clusters) = 3-5. Individual ancestry denoted by color.

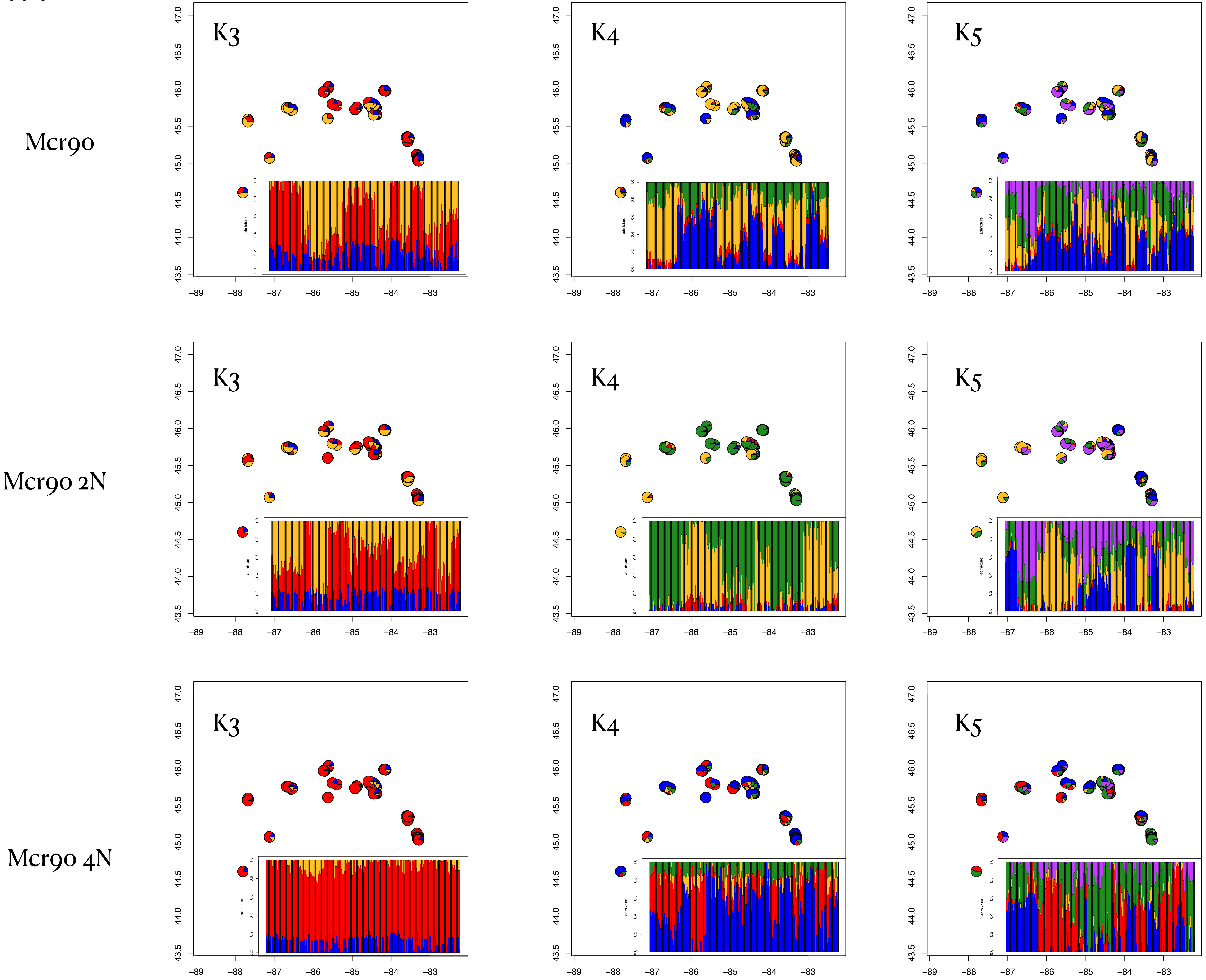

Figure S6. Results for best K from StructureSelector for analyses with fastStructure for (A) MCR90 all loci, (B) MCR90 diploid loci, and (C) MCR90 tetraploid loci.

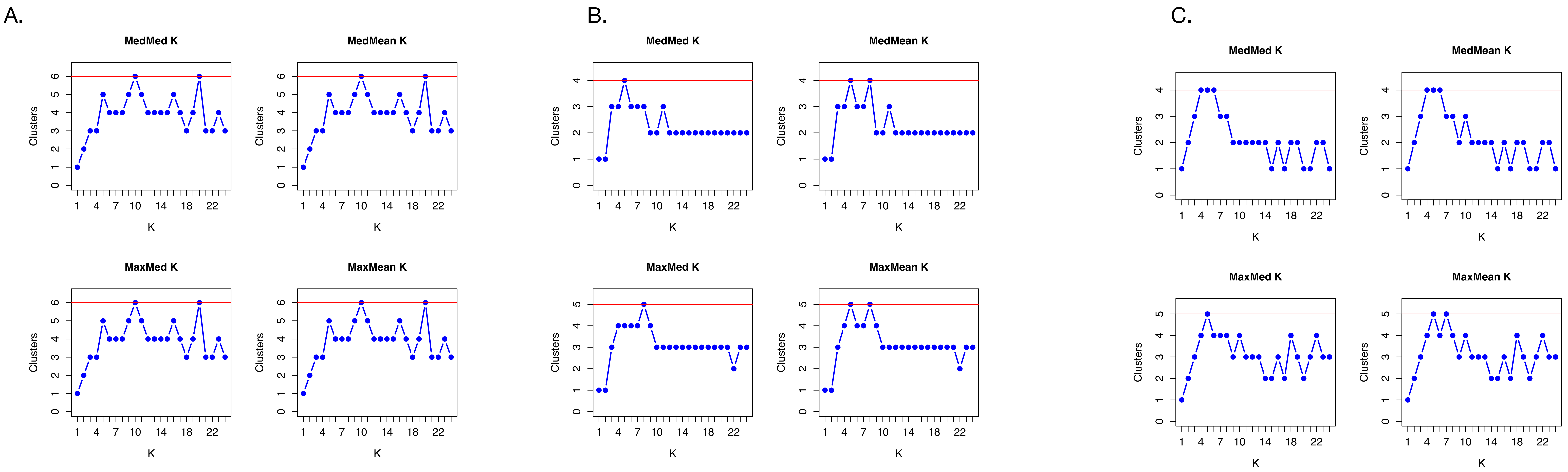

Figure S7. Results for best K from StructureSelector for analyses with fastStructure for (A) MCR50 all loci, (B) MCR50 diploid loci, and (C) MCR50 tetraploid loci.

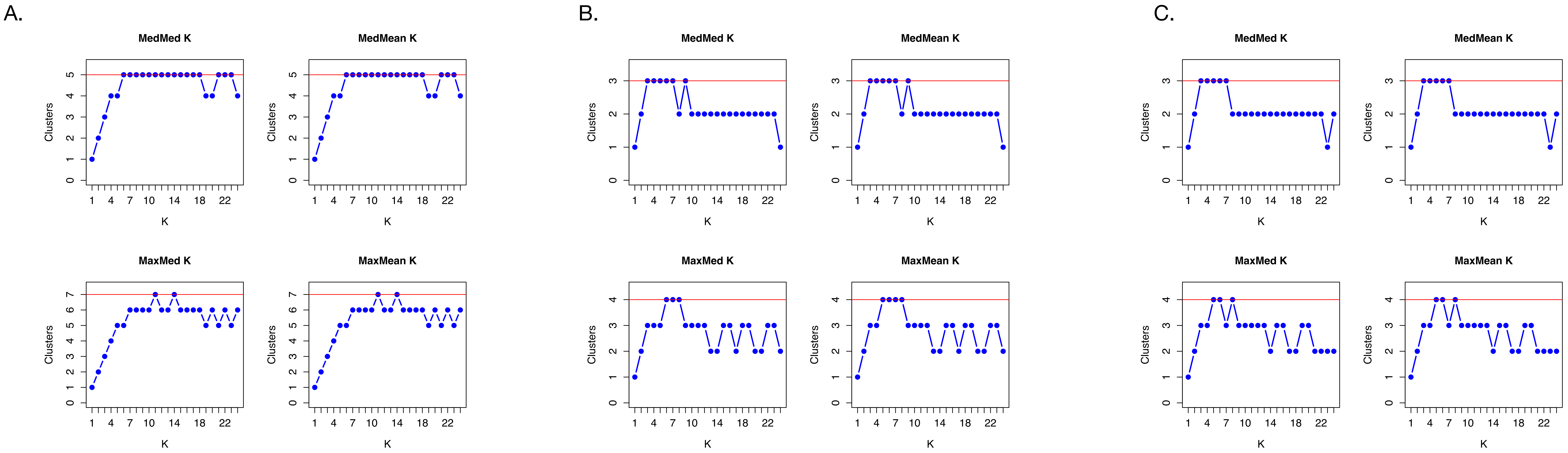

Figure S8. Results for best K from StructureSelector for analyses with Structure without admixture for (A) MCR90 all loci, (B) MCR90 diploid loci, and (C) MCR90 tetraploid loci.

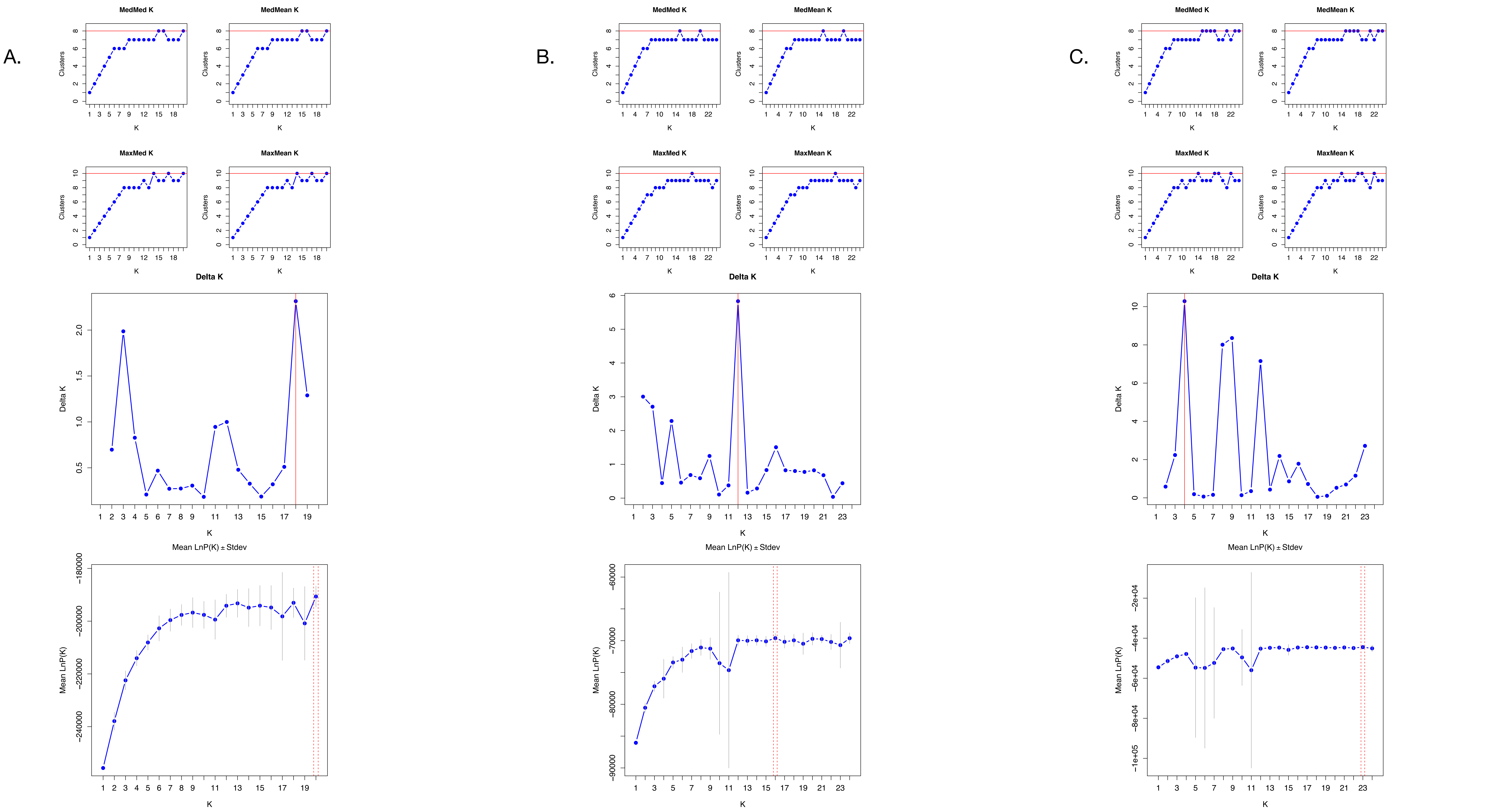

Figure S9. Results for best K from StructureSelector for analyses with Structure with admixture for (A) MCR90 all loci, (B) MCR90 diploid loci, and (C) MCR90 tetraploid loci.

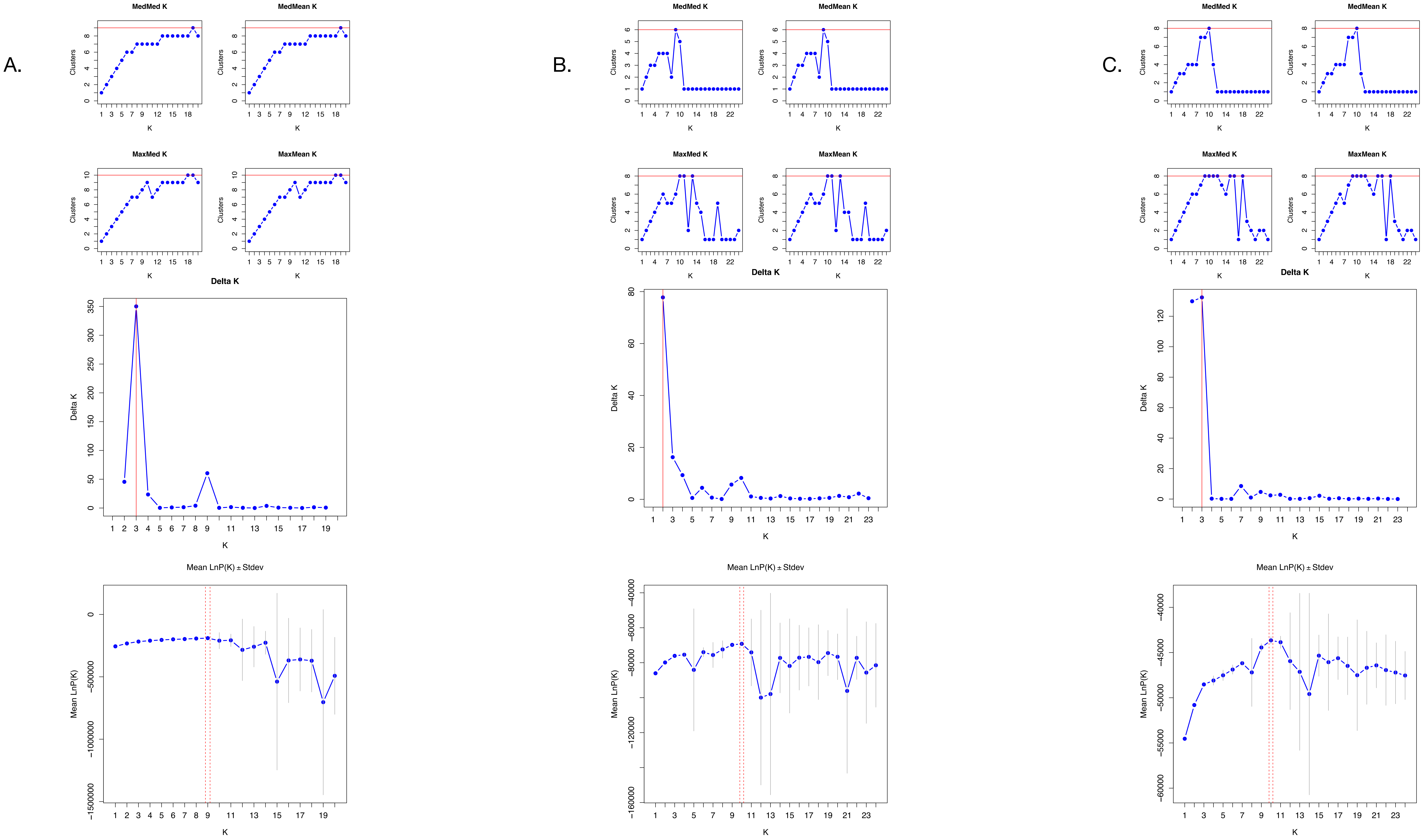

Figure S10. Results for best K from MavericK, based on thermodynamic integration (TI), for analyses without admixture (A) MCR90 all loci, (B) MCR90 diploid loci, and (C) MCR90 tetraploid loci.

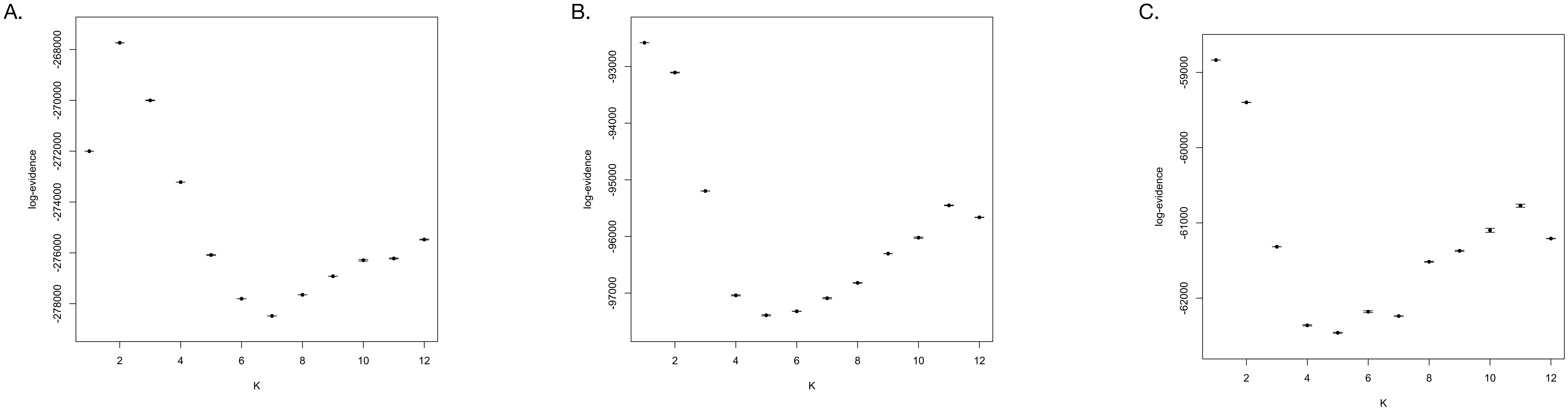

Figure S11. Results for cross-validation scores for tess3r analyses for (A) MCR90 all loci, (B) MCR90 diploid loci, (C) MCR90 tetraploid loci, (D), MCR50 all loci, (E) MCR50 diploid loci, and (F) MCR50 tetraploid loci.

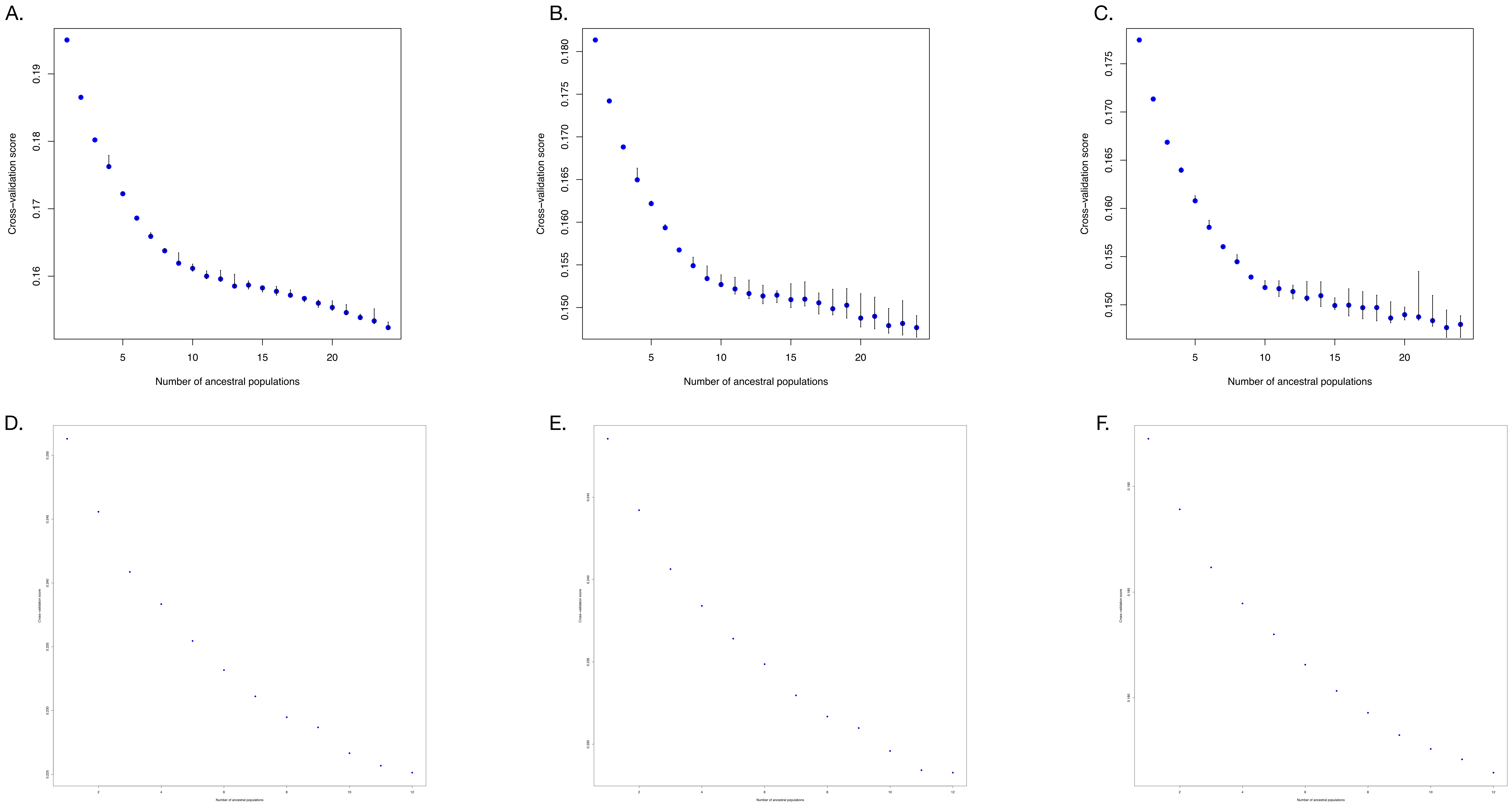

Figure S12. Results for cross-validation scores for conStruct validation analyses for (A) MCR90 all loci, (B) MCR90 diploid loci, and (C) MCR90 tetraploid loci to identify best K (clusters). Graphs with blue and green dots are for spatial and non-spatial models, respectively, and graph with only blue dots displays predictive accuracy for spatial model with confidence intervals.

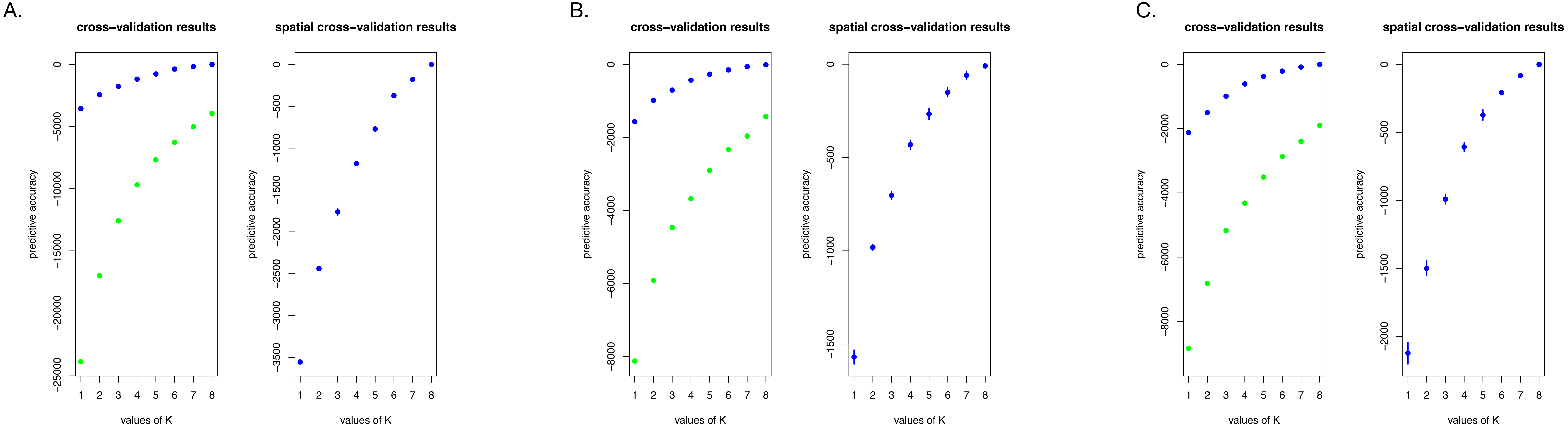

Figure S13. Results for Bayesian Information Criterion (BIC), to identify best K (clusters), from discriminant analysis of principal components (DAPC) for (A) MCR90 all loci, (B) MCR90 diploid loci, (C) MCR90 tetraploid loci, (D), MCR50 all loci, (E) MCR50 diploid loci, and (F) MCR50 tetraploid loci.

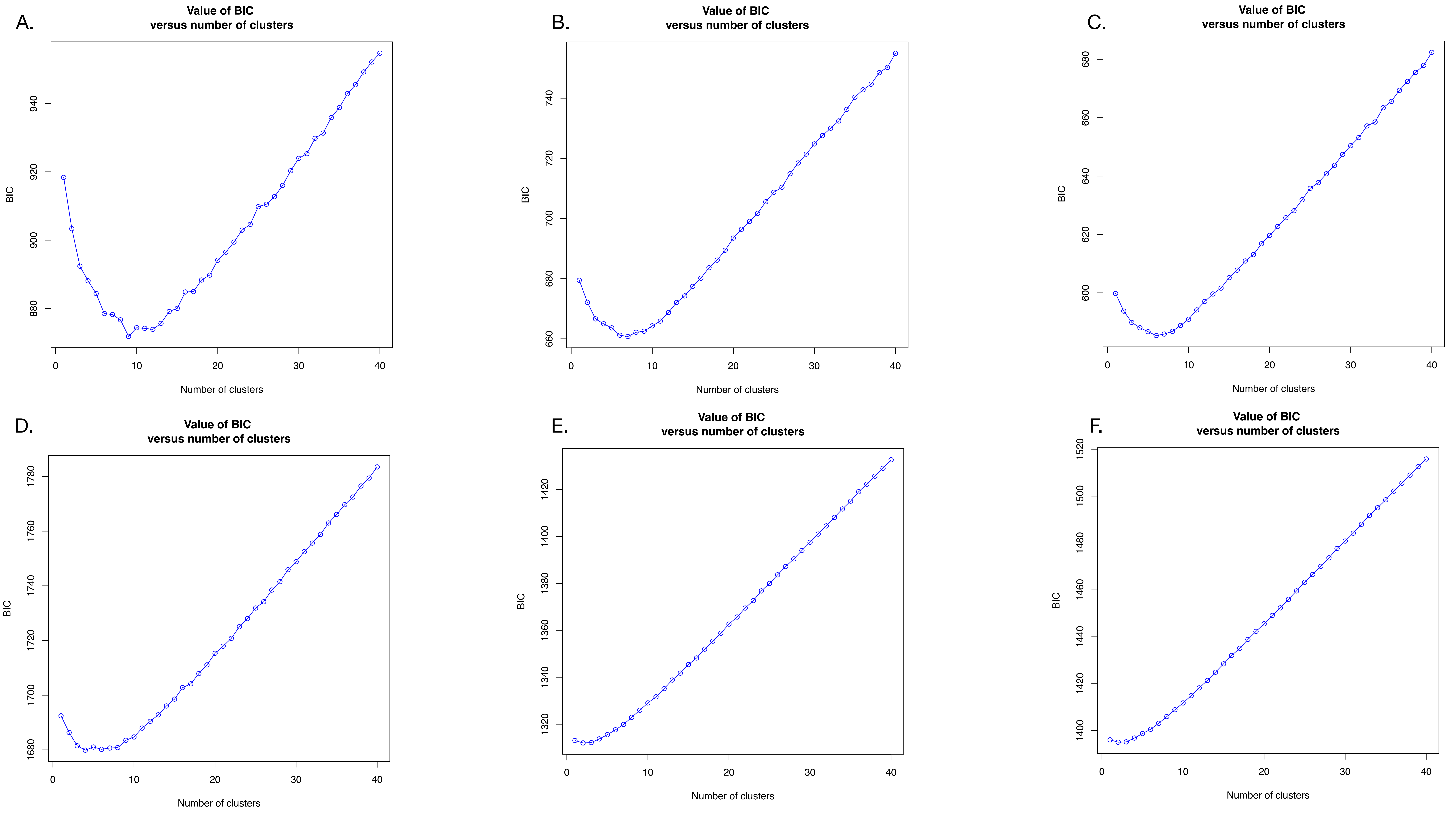

Figure S14. Results from StructureSelector for best K (clusters) fastStructure analyses for loci under selection for (A) MCR90 all loci, (B) MCR50 all loci, (C) MCR50 diploid loci, and (D) MCR50 tetraploid loci.

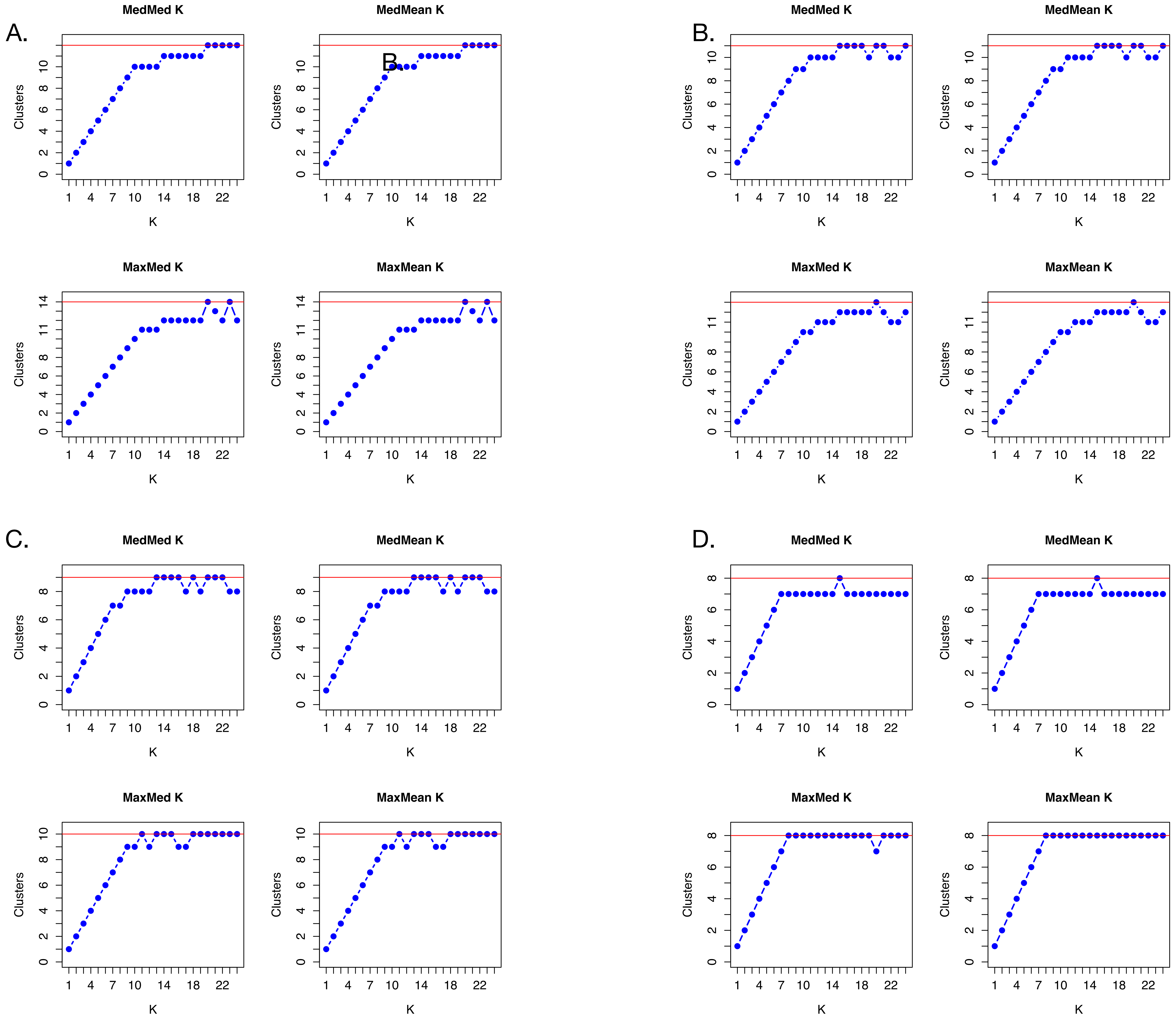

Figure S15. Results from StructureSelector for best K (clusters) fastStructure analyses for loci not under selection for (A) MCR90 all loci, (B) MCR50 all loci, (C) MCR50 diploid loci, and (D) MCR50 tetraploid loci.

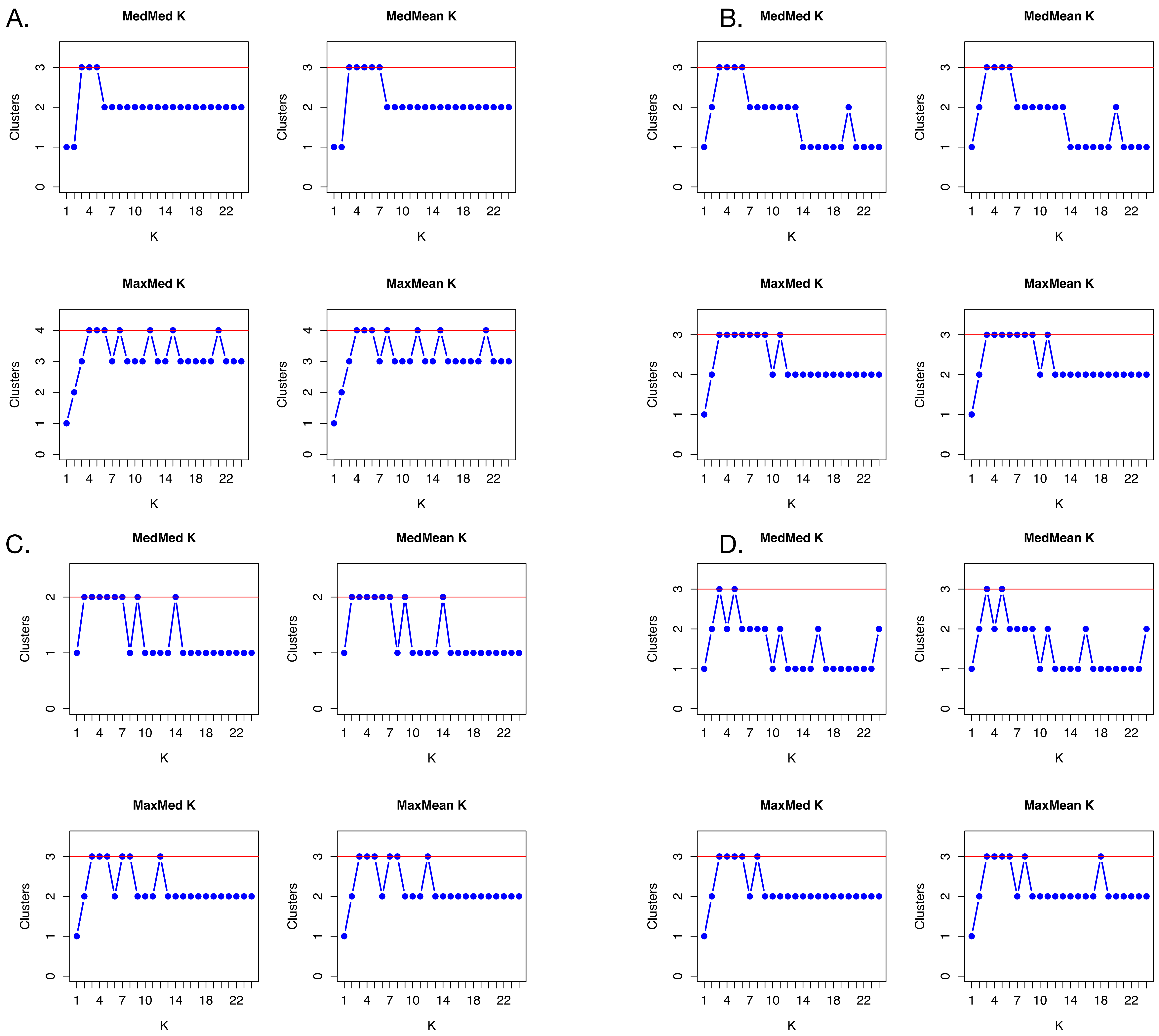

Figure S16. Results for Bayesian Information Criterion (BIC), to identify best K (clusters), from discriminant analysis of principal components (DAPC) analyses for (A) MCR90 all loci under selection, (B) MCR50 all loci under selection, (C) MCR50 diploid loci under selection, (D), MCR50 tetraploid loci under selection, (E) MCR90 all loci not under selection, (F) MCR50 all loci not under selection, (G) MCR50 diploid loci not under selection, (H), MCR50 tetraploid loci not under selection.

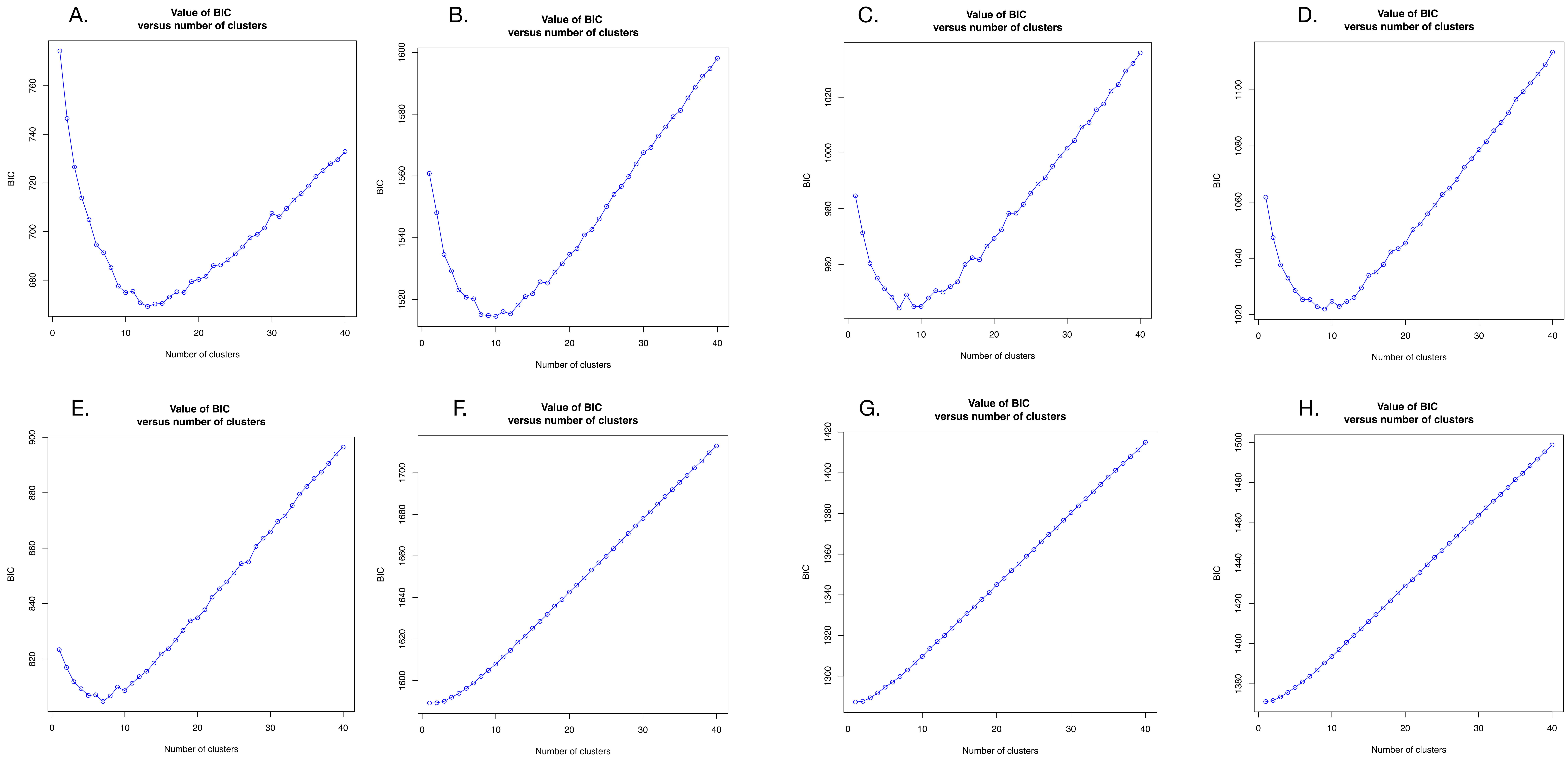

Figure S17. 15 branching scenarios evaluated in DIYABC. Pop 1 is East, Pop 2 is Mid 1, Pop 3 is Mid 2, Pop 4 is West. See Table 1 for population assignation to each population. Change in color represents potential change in population size. Scenario 3 is optimal for all and tetraploid loci, and scenario 7 is optimal for diploid loci.

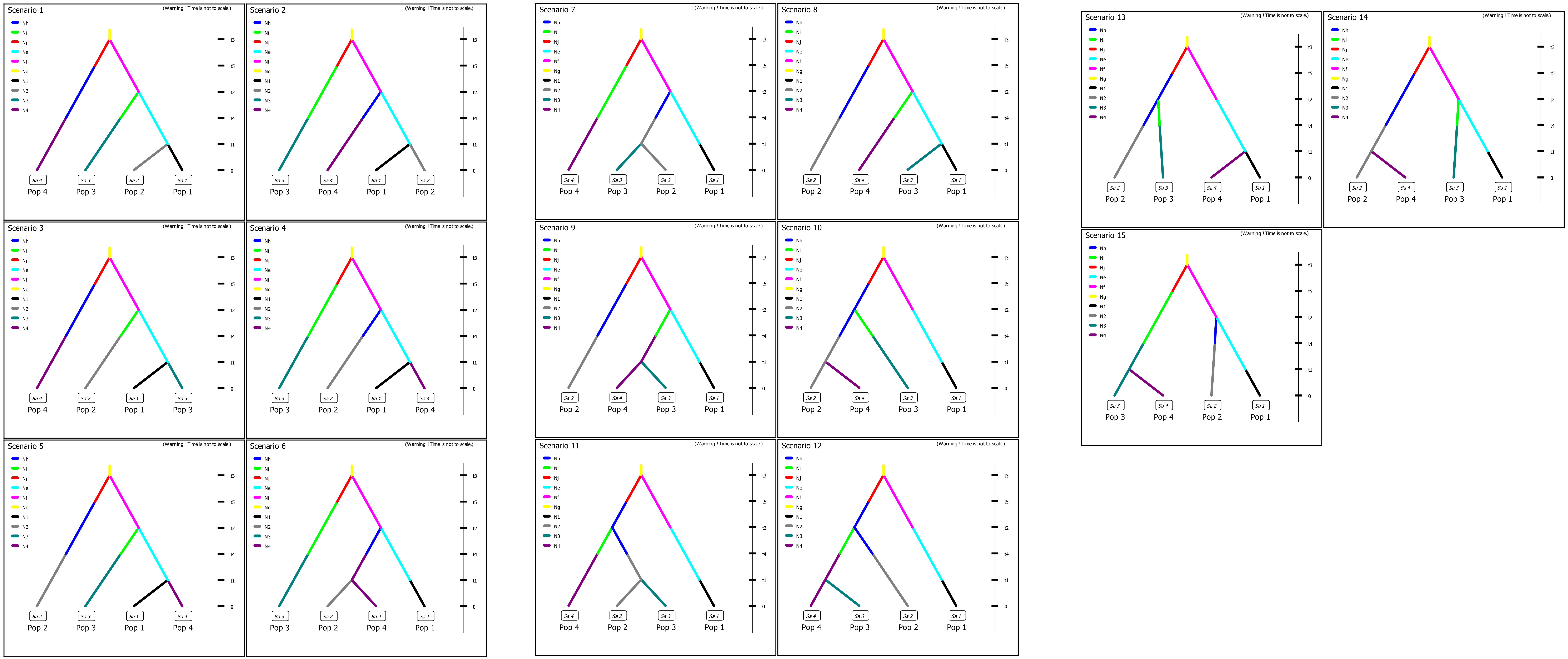

Figure S18. Nine Adaptive Units recognized from population genetic analyses using loci under selection. Map of locations sampled in present study. Dark gray entire lines denote division between East, Mid1, Mid2, and West clusters (also recognized as Management Units). The dashed gray line separates Mid1 and Mid2 populations, and Mid includes both groups of populations together. Light gray lines separate Wisconsin (USA), Michigan (USA), and Ontario (Canada). Scale bar, in red, represents 50 kilometers.

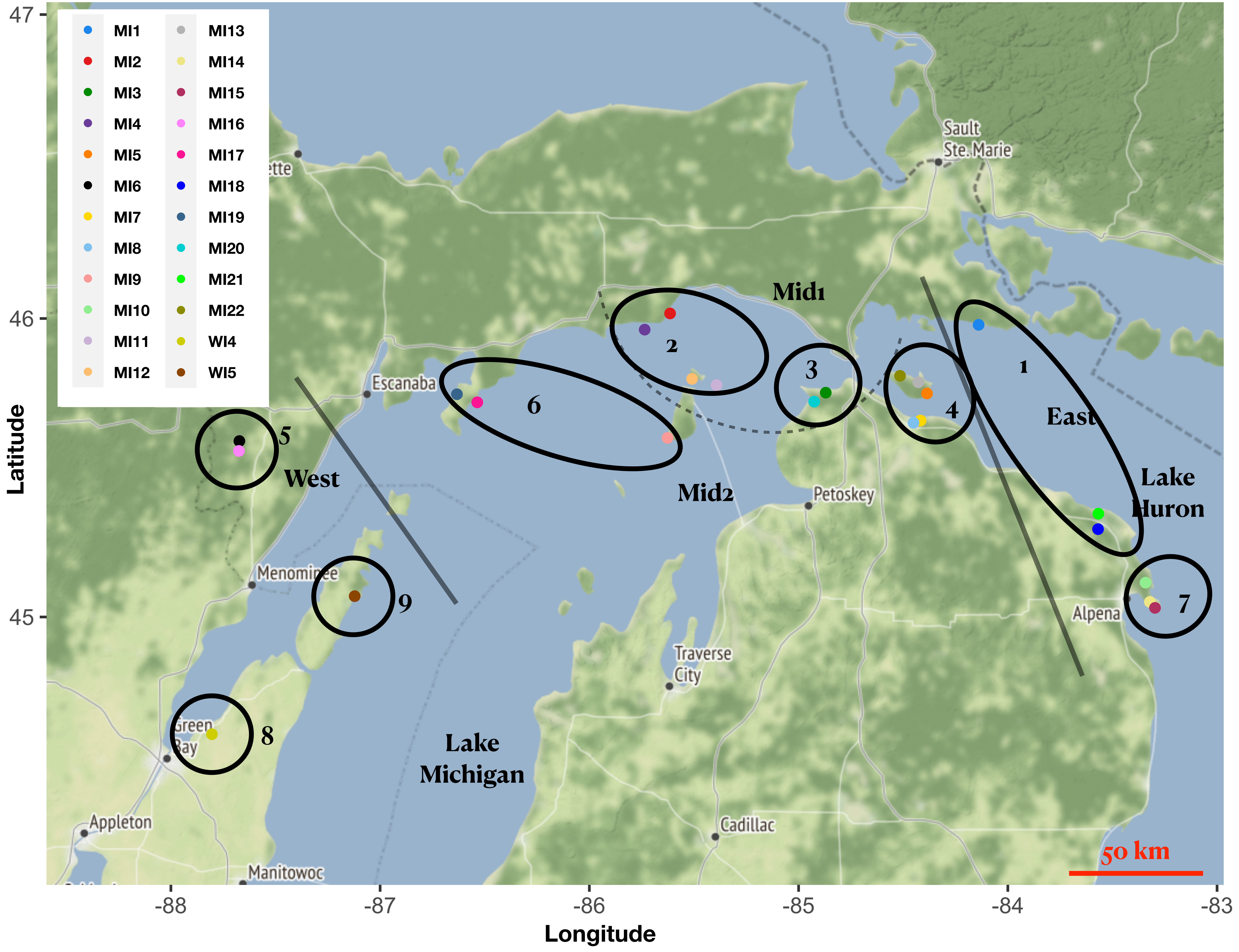

Supplement: Supplementary file 1 [file plants-12-02557-s001.zip › SUPPLEMENTAL FIGURES new names.pdf]
