# Supplementary material for: The Conservation Genetics of Iris lacustris (Dwarf Lake Iris), a Great Lakes Endemic
Source: Plants (Basel). 2023 Jul 5;12(13):2557. doi: 10.3390/plants12132557 (PMC10346457; doi:10.3390/plants12132557)
Supplement: Supplementary file 1 [file plants-12-02557-s001.zip › Supplemental Table.pdf]

Supplemental Table S1. AMOVA results for all datasets.

MCR90 24 populations	Df	Sum Sq	Mean Sq	Sigma	Percentage	Alter	Pvalue
Between POP	23	5423.733	235.81449	14.877835	28.88499	less	0.001
Between samples Within POP	145	4154.267	28.65012	-7.979196	-15.49143	greater	1
Within samples	169	7538.839	44.60851	44.608512	86.60645	greater	0.001
Total	337	17116.839	50.79181	51.50715	100		

MCR90 3 populations							
Between POP	2	1865.921	932.96036	8.1645148	15.204525	less	0.001
Between samples Within POP	166	7712.08	46.45831	0.9249003	1.722413	greater	0.126
Within samples	169	7538.839	44.60851	44.608512	83.073061	greater	0.001
Total	337	17116.839	50.79181	53.6979271	100		

MCR90 4 populations							
Between POP	3	2363.612	787.87082	9.0284928	16.9725958	less	0.001
Between samples Within POP	165	7214.388	43.72356	-0.4424736	-0.8318029	greater	0.684
Within samples	169	7538.839	44.60851	44.608512	83.859207	greater	0.001
Total	337	17116.839	50.79181	53.1945312	100		

MCR90 diploid 24 populations							
Between POP	23	1506.979	65.52083	3.942773	21.54901	less	0.429
Between samples Within POP	145	1539.933	10.62022	-3.733774	-20.40674	greater	1
Within samples	169	3056.834	18.08777	18.087773	98.85773	greater	0.001
Total	337	6103.745	18.112	18.296772	100		

MCR90 diploid 3 populations	2	493.3479	246.67397	2.130147	11.291234	less	0.443
Between POP	166	2553.5637	15.38291	-1.352429	-7.168801	greater	1
Between samples Within POP	169	3056.8336	18.08777	18.087773	95.877567	greater	0.001
Within samples	337	6103.7452	18.112	18.86549	100		
Total							

MCR90 diploid 4 populations

Between POP	3	612.4799	204.15998	2.297999	12.276322	less	0.438
Between samples Within POP	165	2434.4317	14.75413	-1.666821	-8.904455	greater	1
Within samples	169	3056.8336	18.08777	18.087773	96.628133	greater	0.001
Total	337	6103.7452	18.112	18.718951	100		

MCR90 diploid 24 populations

Between POP	23	911.1029	39.61317	2.33995	20.60998	less	0.072
Between samples Within POP	145	1019.4748	7.03086	-1.982671	-17.46311	greater	1
Within samples	169	1858.3582	10.9962	10.996202	96.85314	greater	0.001
Total	337	3788.9359	11.24313	11.353481	100		

MCR90 diploid 3 populations

Between POP	2	280.0636	140.031787	1.1980942	10.268537	less	0.055
Between samples Within POP	166	1650.5141	9.942856	-0.5266732	-4.513972	greater	1
Within samples	169	1858.3582	10.996202	10.9962024	94.245434	greater	0.001
Total	337	3788.9359	11.243133	11.6676234	100		

MCR90 diploid 4 populations

Between POP	3	358.1321	119.377365	1.3327421	11.493288	less	0.06
Between samples Within POP	165	1572.4456	9.529973	-0.7331146	-6.322226	greater	1
Within samples	169	1858.3582	10.996202	10.9962024	94.828938	greater	0.001
Total	337	3788.9359	11.243133	11.5958299	100		
MCR50 24 populations							
Between POP	23	5423.733	235.81449	14.877835	28.88499	less	0.001
Between samples Within POP	145	4154.267	28.65012	-7.979196	-15.49143	greater	1
Within samples	169	7538.839	44.60851	44.608512	86.60645	greater	0.001
Total	337	17116.839	50.79181	51.50715	100		
MCR50 3 populations							
Between POP	2	1865.921	932.96036	8.1645148	15.204525	less	0.001
Between samples Within POP	166	7712.08	46.45831	0.9249003	1.722413	greater	0.125
Within samples	169	7538.839	44.60851	44.608512	83.073061	greater	0.001
Total	337	17116.839	50.79181	53.6979271	100		
MCR50 2 populations							
Between POP	3	2363.612	787.87082	9.0284928	16.9725958	less	0.001
Between samples Within POP	165	7214.388	43.72356	-0.4424736	-0.8318029	greater	0.649
Within samples	169	7538.839	44.60851	44.608512	83.859207	greater	0.001
Total	337	17116.839	50.79181	53.1945312	100		
MCR50 diploid 24 populations							
Between POP	23	613.7878	26.686424	1.544482	19.48189	less	0.01
Between samples Within POP	145	751.1724	5.1805	-1.2028	-15.17196	greater	1

Within samples	169	1282.0507	7.586099	7.586099	95.69007	greater	0.001
Total	337	2647.0109	7.854632	7.927781	100		

MCR50 diploid 3 populations

Between POP	2	165.6171	82.808562	0.6961106	8.592219	less	0.011
Between samples Within POP	166	1199.3431	7.224958	-0.1805704	-2.228813	greater	0.889
Within samples	169	1282.0507	7.586099	7.5860991	93.636594	greater	0.001
Total	337	2647.0109	7.854632	8.1016393	100		

MCR50 diploid 4 populations

Between POP	3	213.1698	71.056606	0.7774137	9.644449	less	0.012
Between samples Within POP	165	1151.7904	6.980548	-0.3027757	-3.756179	greater	0.98
Within samples	169	1282.0507	7.586099	7.5860991	94.11173	greater	0.001
Total	337	2647.0109	7.854632	8.0607371	100		

MCR50 tetraploid 24 populations

Between POP	23	1313.55	57.110885	3.492225	22.64801	less	0.685
Between samples Within POP	145	1230.162	8.483878	-3.443463	-22.33178	greater	1
Within samples	169	2597.666	15.370805	15.370805	99.68377	greater	0.001
Total	337	5141.379	15.256316	15.419566	100		

MCR50 tetraploid 3 populations

Between POP	2	460.9228	230.4614	2.006951	12.570298	less	0.661
Between samples Within POP	166	2082.7899	12.54693	-1.411939	-8.843511	greater	1
Within samples	169	2597.666	15.3708	15.370805	96.273213	greater	0.001
Total	337	5141.3786	15.25632	15.965816	100		

MCR50 tetraploid 4 populations

Between POP	3	580.4313	193.47711	2.203031	13.90998	less	0.709
Between samples Within POP	165	1963.2813	11.89867	-1.736065	-10.96155	greater	1
Within samples	169	2597.666	15.3708	15.370805	97.05157	greater	0.001
Total	337	5141.3786	15.25632	15.837771	100		

Supplemental Table S2. K values for the MCR90 datasets for STRUCTURE and Maverick.

	No admixture			Admixture			
	STRUCTURE (Evano et al. (2005)	Ln Pr(X|K)	StructureSelector	STRUCTURE (Evano et al. (2005)	Ln Pr(X|K)	StructureSelector	MaverickK
MCR90	18	20	8-10	3	9	9-10	2
MCR90 diploid loci	12	16	8-10	2	10	6-8	1
MCR90 tetraploid loci	4	23	8-10	3	10	8	1
